# Recent advances in organocatalytic atroposelective reactions

**DOI:** 10.3762/bjoc.21.6

**Published:** 2025-01-09

**Authors:** Henrich Szabados, Radovan Šebesta

**Affiliations:** 1 Department of Organic Chemistry, Faculty of Natural Science, Comenius University Bratislava, Mlynská dolina, Ilkovičova 6, 842 15 Bratislava, Slovakia

**Keywords:** asymmetric organocatalysis, atropoisomers, atroposelective synthesis, axial chirality, stereogenic axis

## Abstract

Axial chirality is present in a variety of naturally occurring compounds, and is becoming increasingly relevant also in medicine. Many axially chiral compounds are important as catalysts in asymmetric catalysis or have chiroptical properties. This review overviews recent progress in the synthesis of axially chiral compounds via asymmetric organocatalysis. Atroposelective organocatalytic reactions are discussed according to the dominant catalyst activation mode. For covalent organocatalysis, the typical enamine and iminium modes are presented, followed by *N*-heterocyclic carbene-catalyzed reactions. The bulk of the review is devoted to non-covalent activation, where chiral Brønsted acids feature as the most prolific catalytic structure. The last part of the article discusses hydrogen-bond-donating catalysts and other catalyst motifs such as phase-transfer catalysts.

## Introduction

Stereoselective catalytic formation of chiral compounds is one of the critical tasks of modern organic synthesis [1]. The catalytic formation of compounds with a center of chirality has been the focus of countless works and can now be considered a matured area. On the other hand, the generation of compounds comprising a stereogenic plane or axis is much less developed. Axially chiral compounds are well known as chiral ligands in asymmetric catalysis, with notable examples of binaphthyl-based derivatives such as BINAP, SEGPHOS, or binaphthyl-based phosphoric acid derivatives, which are among the privileged catalyst frameworks [2]. Axially chiral biaryls have also been found to be useful in materials [3]. Although much less widely occurring than centrochiral compounds, there are also naturally occurring axially chiral compounds. Axially chiral compounds are becoming increasingly relevant also in drug discovery and medicine [4]. However, a lack of reliable synthetic methods for their preparation hindered the broader application of axially chiral compounds. In recent decades, there has been increased interest in the catalytic syntheses of axially chiral compounds by catalytic [5–6], especially organocatalyzed, methods [7–11]. Asymmetric organocatalysis offers efficient and environmentally benign access to numerous chiral compounds [12]. Therefore, an increasing number of researchers are now investigating the organocatalytic formation of compounds with axial stereogenic axes across various structural motifs [13–17]. Remarkably, these compounds are not limited to the C(sp^2^)–C(sp^2^) axis, but new developments in the formation of C–N, C–B, or even N–N stereogenic axes have been achieved. To help the research community distill and abstract the relevant new knowledge, this review has been prepared, which aims to provide an update on the last five years of this burgeoning area with some relevant links to key earlier works. The material in this article is divided according to the major activation mode of the organocatalyst, from covalent activation via enamine and iminium activation to NHC-catalyzed reactions. The major part is devoted to chiral Brønsted acid catalysis as it seems so far the most widely used activation principle for the generation of axially chiral compounds. Hydrogen-bond-donating catalysts and various other activation modes complete the discussion of recent advances in organocatalytic atroposelective syntheses.

## Review

### Atroposelective reactions via enamine and iminium activation

**Scheme 1:**
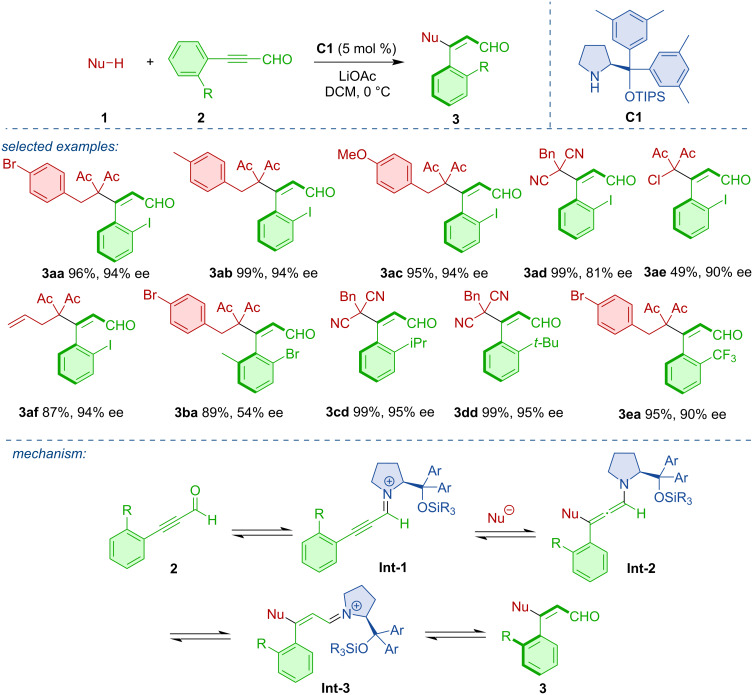
Formation of axially chiral styrenes **3** via iminium activation.

Iminium activation was utilized in the synthesis of axially chiral styrenes. Tan and co-workers developed an atroposelective strategy toward axially chiral alkenylarenes **3** based on an organocatalytic Michael addition to alkynals **2** (Scheme 1) [18]. The authors identified the Jørgensen–Hayashi-type catalyst **C1** as the most efficient organocatalyst. In this way, a range of axially chiral styrenes were obtained in high yields and enantiomeric purities. The reaction was based on an iminium activation of propargylic aldehydes with catalyst **Int-1**. Another critical feature was the ability of the organocatalyst to promote the *Z*-selective isomerization of **Int-2** to **Int-3**.

**Scheme 2:**
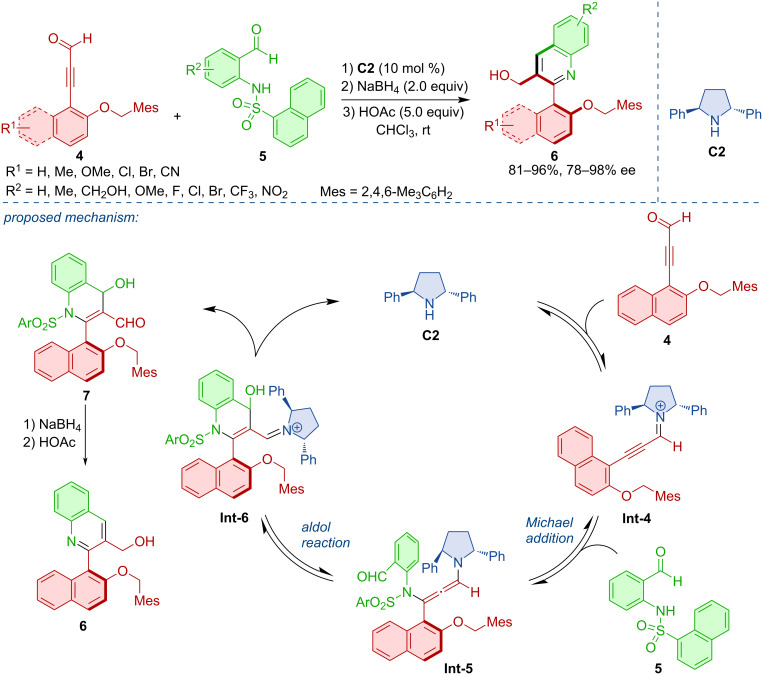
Synthesis of axially chiral 2-arylquinolines **6**.

In a related fashion, Wang and co-workers developed an atroposelective heterocycloaddition [19]. The iminium-activated alkynals **4** reacted with aminoarylaldehydes **5** to form axially chiral 2-arylquinoline derivatives **6** (Scheme 2). Using the pyrrolidine derivative **C2** as the most efficient organocatalyst, a range of quinoline derivatives were obtained in high yields and enantiomeric purities. The postulated mechanism consists of iminium activation, atroposelective aza-Michael addition, and intramolecular aldol reaction to form the cationic intermediate **Int-6**. Release of the catalyst **C2**, reduction with NaBH_4_, and dehydration with acetic acid leads to the desired product **6**.

**Scheme 3:**
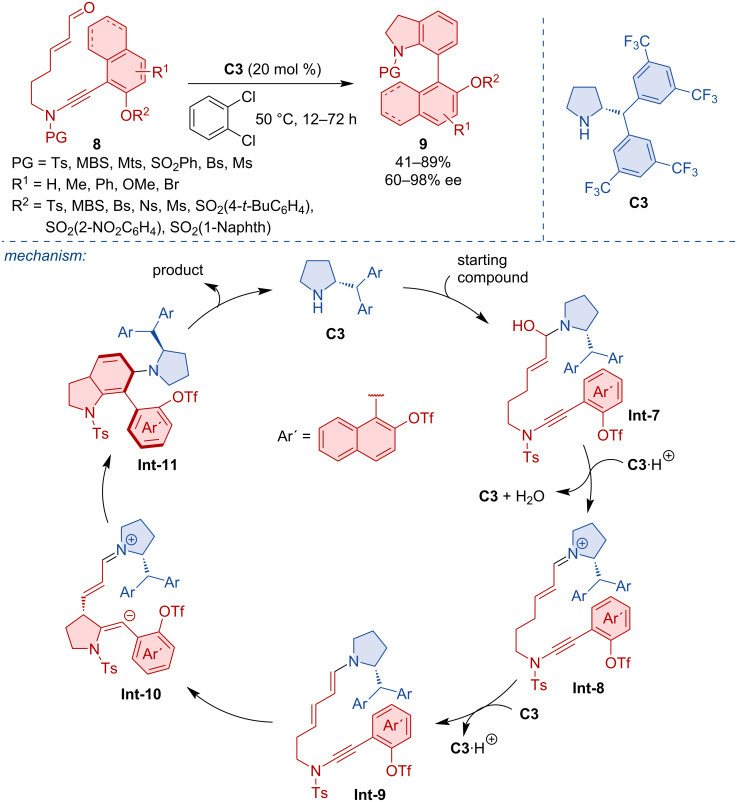
Atroposelective intramolecular (4 + 2) annulation leading to aryl-substituted indolines.

Recently, an organocatalytic atroposelective intramolecular (4 + 2) annulation of enals with ynamides **8** to afford axially chiral 7-arylindolines **9** was reported [20]. The reaction mechanism, rationalized by DFT calculations, is believed to occur through catalyst **C3** activation of the substrate **8**, dehydration, and deprotonation with tautomerization leading to the enamine intermediate **Int-9**. As the assumed rate-determining step the intramolecular nucleophilic addition takes place, followed by further cyclization and finally, release of the organocatalyst to form the axially chiral product **9**. Various aryl-substituted indolines **9** were obtained in good yields and high enantiomeric purities (Scheme 3).

**Scheme 4:**
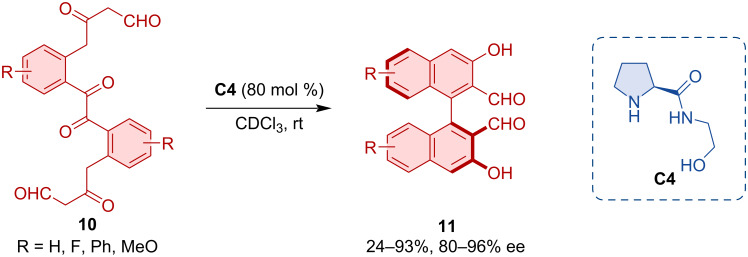
Atroposelective formation of biaryl via twofold aldol condensation.

Sparr and co-workers developed an atroposelective synthesis for tetra-*ortho*-substituted biaryls **11** by non-canonical polyketide cyclizations [21]. This work was based on an earlier report of the team on the aldol cyclization of naphthyl-substituted unsaturated ketoaldehydes [22]. The process was inspired by the biocatalytic synthesis of aromatic polyketides by polyketide synthase from poly β-carbonyl substrates. Pyrrolidine-based organocatalyst **C4** was able to promote a twofold atroposelective arene-forming 6-*enolendo* aldol condensation (Scheme 4).

**Scheme 5:**
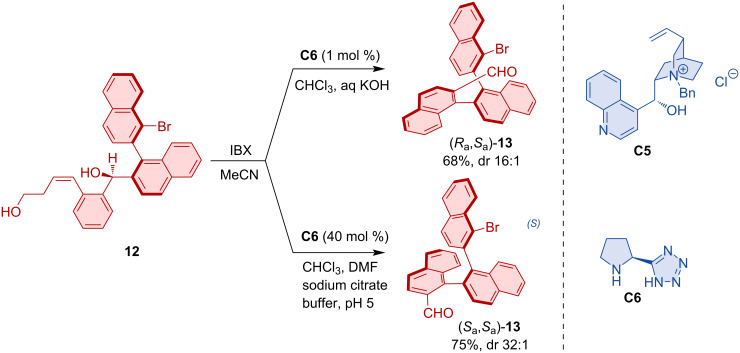
Strategy towards diastereodivergent formation of axially chiral oligonaphthylenes.

Sparr also realized a central-to-axial chirality conversion via catalyst-controlled oxidative aromatization [23]. In this way, the axially chiral starting material **12** comprising an additional stereogenic center was converted into oligonaphthylenes **13** with two, three or even four stereogenic axes. Based on the organocatalyst used, the transformation produced either the (*R*_a_,*S*_a_)-isomer using pyrrolidine tetrazole catalyst **C6** or the (*S*_a_,*S*_a_)-diastereoisomer using quaternary ammonium salt **C5** (Scheme 5). Catalyst-controlled formation of twofold and higher-order stereogenicity in axially chiral arenes was discussed in this account article [15].

**Scheme 6:**
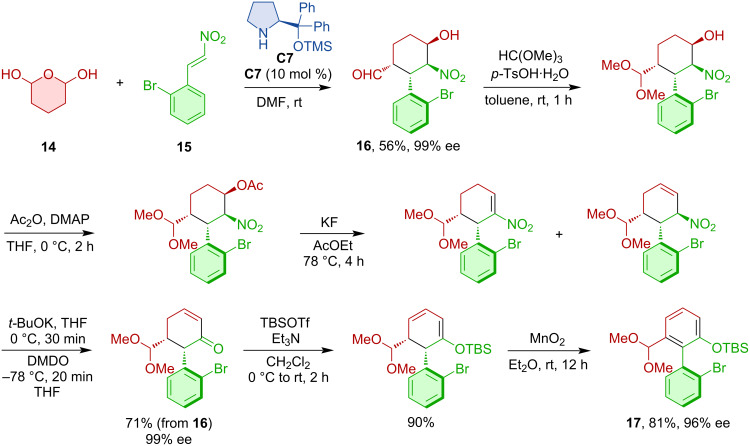
Atroposelective formation of chiral biaryls based on a Michael/Henry domino reaction.

Hayashi realized an organocatalytic domino sequence that afforded axially chiral biaryls [24]. The transformation relied on an organocatalytic Michael/Henry cascade. The enamine-type Michael addition was catalyzed by the Hayashi–Jørgensen organocatalyst **C7** (Scheme 6). Then, a series of one-pot reactions was carried out to provide the final biaryl products **17**.

**Scheme 7:**
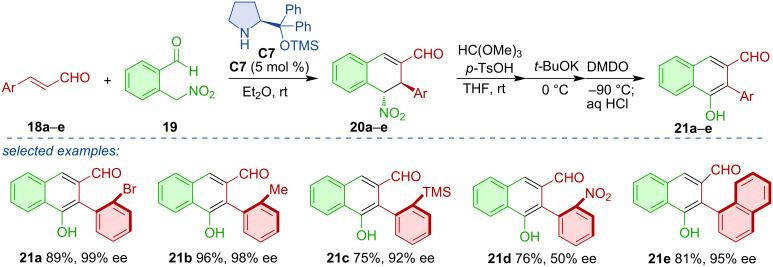
Organocatalytic Michael/aldol cascade followed by oxidative aromatization.

In a related strategy, Hayashi´s team realized an organocatalytic Michael/aldol cascade leading to chiral dihydronaphthalene derivatives **20a**–**e** [25]. Through a series of one-pot reactions, aromatization was achieved with concomitant central-to-axial chirality conversion and formation of axially chiral products **21a**–**e** (Scheme 7). This critical aromatization was later studied in more detail, and the team was able to achieve enantiodivergent aromatization, which led to different atropoisomers based on the oxidation reagent used [26]. The use of NBS and AgOTf led to the formation of the (*S*_a_)-atropoisomers, whereas NIS afforded the (*R*_a_)-atropoisomers.

**Scheme 8:**
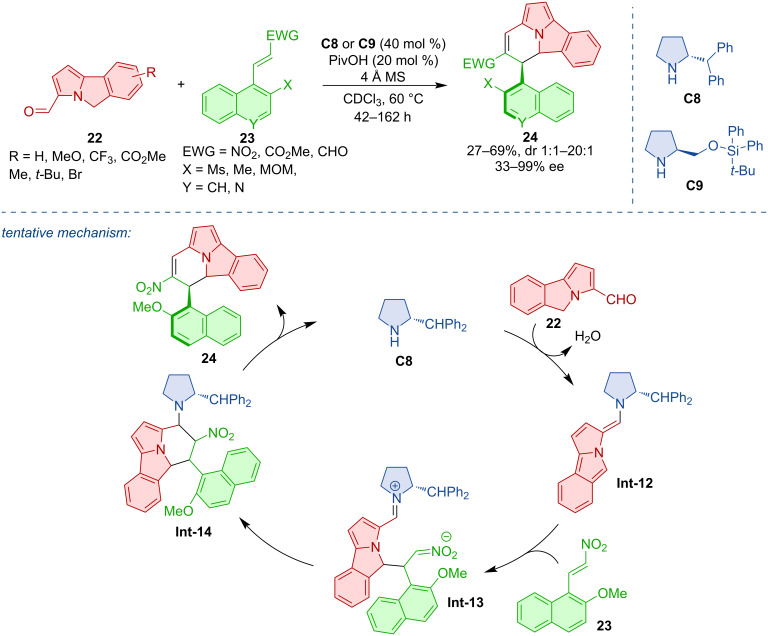
Atroposelective formation of C(sp^2^)–C(sp^3^) axially chiral compounds.

Non-biaryl atropoisomers are characterized by at least one non-aryl substituent on the stereogenic axis. Among them, compounds featuring a conformationally stable C(sp^2^)–C(sp^3^) stereogenic axis are of interest and have been recently investigated by Jørgensen and co-workers. The authors employed an enantioselective aminocatalytic cycloaddition between 5*H*-benzo[*a*]pyrrolizine-3-carbaldehydes **22** and naphthyl-substituted nitroalkenes, α,β-unsaturated ketoesters, or α,β-unsaturated aldehydes **23** [27]. This transformation led to a series of axially chiral cycl[3.2.2]azines **24** in good yields and high enantiomeric purities (Scheme 8). The proposed mechanism comprises enamine activation, condensation with nitroolefin **23**, ring closure, and catalyst elimination to provide the axially chiral product **24**.

### NHC-catalyzed atroposelective reactions

Organocatalysis with *N*-heterocyclic carbenes (NHC) became one of the main types of covalent activation strategies, which grew into a very diverse area, allowing the synthesis of a wide array of interesting structures. Also, in atroposelective synthesis, NHC-catalysis recently led to an array of intriguing transformations.

**Scheme 9:**
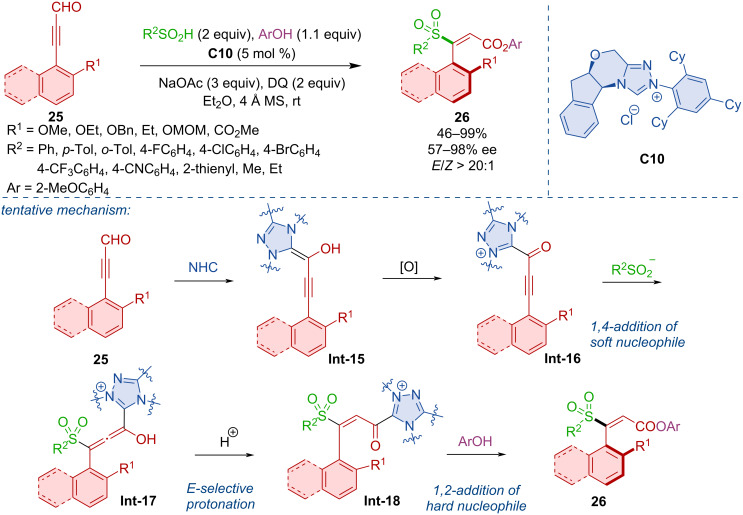
NHC-catalyzed synthesis of axially chiral styrenes **26**.

Axially chiral styrenes **26** were assembled via the NHC-catalyzed reaction of propargylic aldehydes **25**, sulfinic acids, and phenols [28]. The crucial step of this transformation is the 1,4-addition of the sulfinic anion to the triple bond of acylazolium intermediate **Int-16** followed by *E*-selective protonation of **Int-17** (Scheme 9).

**Scheme 10:**
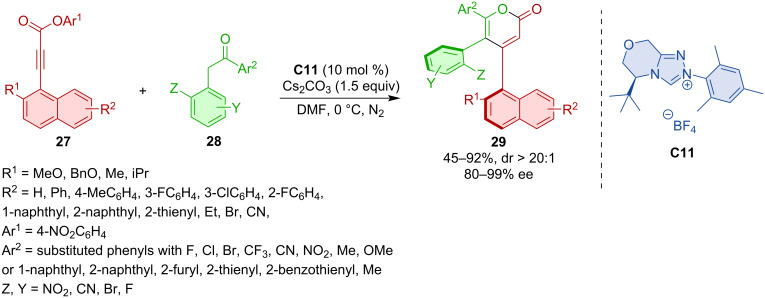
NHC-catalyzed synthesis of biaxial chiral pyranones.

NHC-catalysis also proved useful in the atroposelective construction of triaryl derivatives with two stereogenic axes. Wei, Du, and co-workers developed a synthesis of 1,2-diaxially chiral triarylpyranones **29** via an NHC-catalyzed (3 + 3) annulation [29]. A broad scope was demonstrated, comprising more than 50 diversely substituted compounds (Scheme 10).

**Scheme 11:**
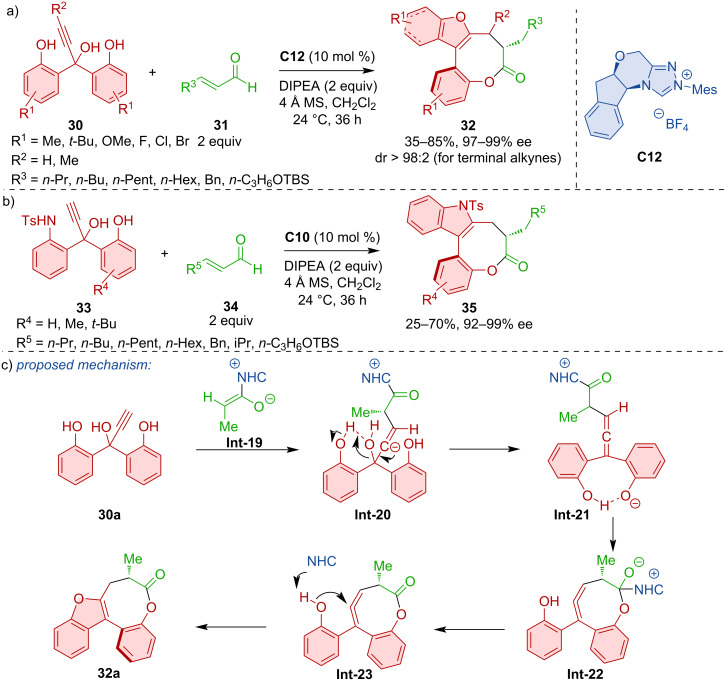
Formation of bridged biaryls with eight-membered lactones.

Wong, Zhao, and co-workers disclosed the intriguing formation of bridged biaryls featuring eight-membered lactone rings **32** [30]. This serendipitously discovered transformation relies on the catalysis with azolium precatalyst **C12** (Scheme 11a). The reaction also allowed the synthesis of indol-derived bridged biaryls **35** (Scheme 11b). The proposed mechanism, supported by DFT calculations, comprises propargylic substitution towards **Int-20** with NHC-derived enolate **Int-19** followed by lactonization to **Int-21** and **Int-22** (Scheme 11c).

**Scheme 12:**
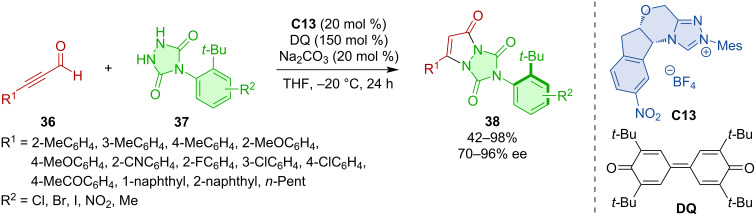
The NHC-catalyzed (3 + 2) annulation of urazoles **37** and ynals **36**.

Chi and co-workers showed that desymmetrization of urazoles can lead to axially chiral derivatives [31]. The NHC-catalyzed (3 + 2) annulation between α,β-unsaturated aldehydes **36** and urazoles **37** generates atropoisomers **38** with a C–N stereogenic axis (Scheme 12).

**Scheme 13:**
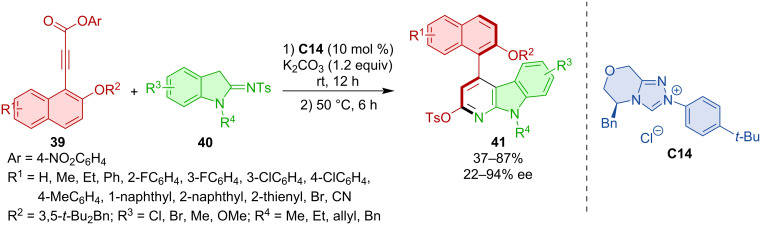
NHC-catalyzed synthesis of axially chiral 4‑aryl α‑carbolines **41**.

Wei, Du, and co-workers developed an atroposelective formal (3 + 3) annulation of 4-nitrophenyl 3-arylpropiolates **39** with 2-sulfonamidoindolines **40** [32]. The NHC catalyst derived from triazolium salt **C14** afforded the best results in terms of chemical yields as well as enantioselectivities (Scheme 13).

**Scheme 14:**
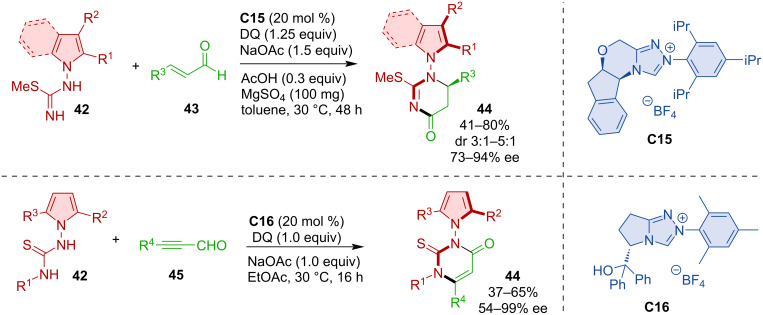
NHC-catalyzed construction of N–N-axially chiral pyrroles and indoles.

Axially chiral compounds with an N–N stereogenic axis can be synthesized by an NHC-catalyzed (3 + 3) annulation [33]. The key feature of this transformation is the cycloaddition of α,β-unsaturated azolium intermediates with thioureas. In this way, a range of diversely substituted N–N axially chiral pyrroles and indoles **44** are obtained (Scheme 14).

**Scheme 15:**
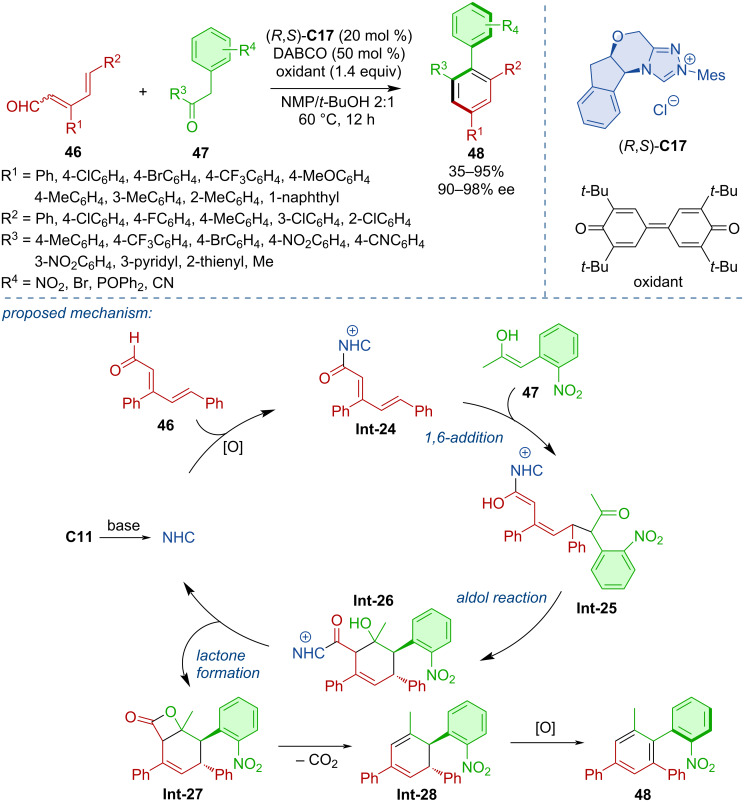
NHC-catalyzed oxidative Michael–aldol cascade.

Zhu and co-workers developed a method for the atroposelective formation of arenes **48** by an NHC-catalyzed formal (4 + 2) cycloaddition [34]. The triazolium pre-catalyst (*R,S*)-**C11** was the most efficient in providing a range of biaryls in high yields and enantiomeric purities. The reaction was initiated by the formation of acylazolium intermediate **Int-24** that underwent a 1,6-addition with the enol form of the carbonyl substrate to give **Int-25**. Cyclization was realized via an intramolecular aldol reaction to **Int-26** (Scheme 15).

**Scheme 16:**
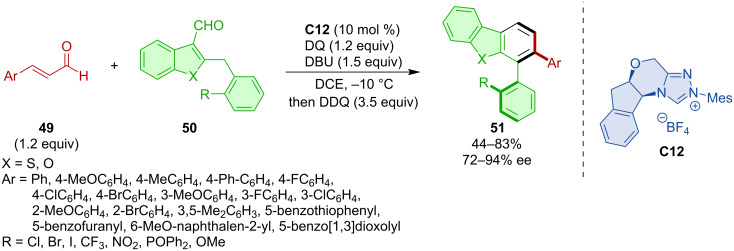
NHC-catalyzed (4 + 2) annulation for the synthesis of benzothiophene-fused biaryls.

Ye and co-workers developed atroposelective formation of benzothiophene-fused biaryls via formal (4 + 2) annulation [35]. The NHC catalyst **C12** was the most efficient for realizing the de novo formation of a new aryl ring within the newly formed axially chiral biaryl **51** from enals **49** and 2-benzylbenzothiophene- or benzofuran-3-carbaldehydes **50** (Scheme 16).

**Scheme 17:**
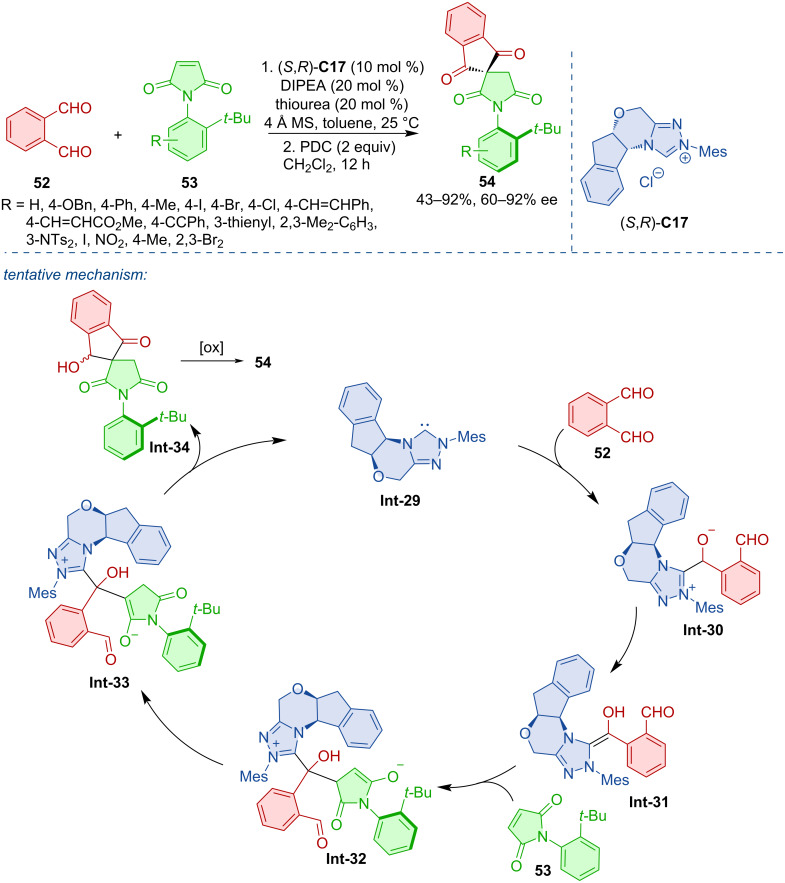
NHC-catalyzed desymmetrization of *N*-aryl maleimides.

Another demonstration of the atroposelective formation of compounds with a C–N stereogenic axis was developed by Jindal, Mukherjee, Biju, and co-workers [36]. The authors developed an NHC-catalyzed desymmetrization of *N*-aryl maleimides **53**, which afforded a range of axially chiral *N*-aryl succinimides **54**. The tentative mechanism comprises the formation of the Breslow intermediate **Int-31** from the catalyst carbene and aldehyde **52**, which then adds to the electron-deficient double bond of maleimide giving rise to **Int-32** (Scheme 17).

**Scheme 18:**
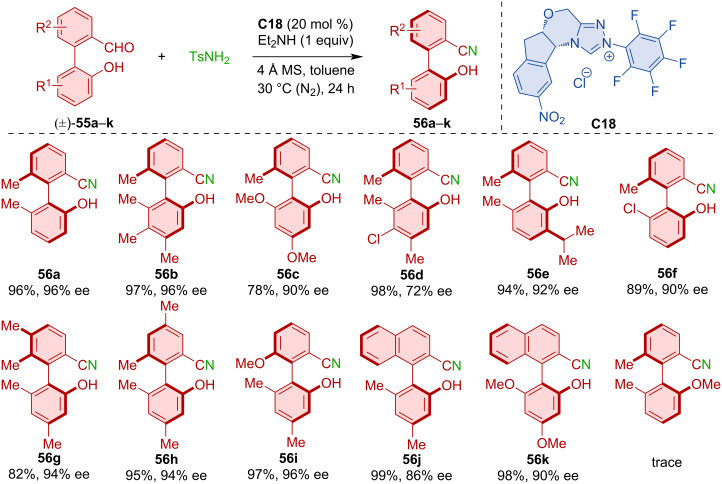
NHC-catalyzed deracemization of biaryl hydroxy aldehydes **55a**–**k** into axially chiral benzonitriles **56a**–**k**.

Chi and co-workers developed an atroposelective deracemization of biaryl hydroxy aldehydes **55a**–**k** [37]. NHC catalyst **C18** afforded a range of axially chiral benzonitriles **56a**–**k** in high yield and enantiomeric purities (Scheme 18). The reaction likely proceeds via the initial formation of racemic imines, which is followed by the formation of aza-Breslow-type intermediates with the chiral NHC-catalyst and subsequent deprotonation toward the nitrile product.

**Scheme 19:**
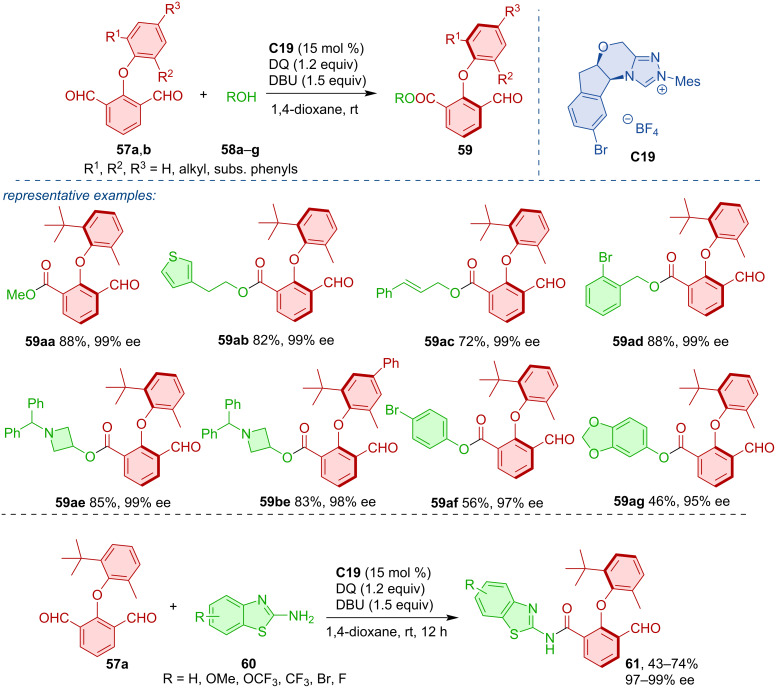
NHC-catalyzed desymmetrization of 2-aryloxyisophthalaldehydes.

Zhang, Wang, Ye, and co-workers utilized NHC-catalysis for the atroposelective synthesis of axially chiral diaryl ethers **59** and **61** [38]. This transformation was realized via desymmetrization of prochiral 2-aryloxyisophthalaldehydes **57a**,**b** with a range of aliphatic and aromatic alcohols **58a**–**g**, as well as heteroaromatic amines **60** (Scheme 19). Chiral diaryl ethers of this type received increased attention lately. Biju, Gao, Zhang, and Zeng groups all reported high degrees of yields and enantioselectivities in similar desymmetrization reactions [39–42].

**Scheme 20:**
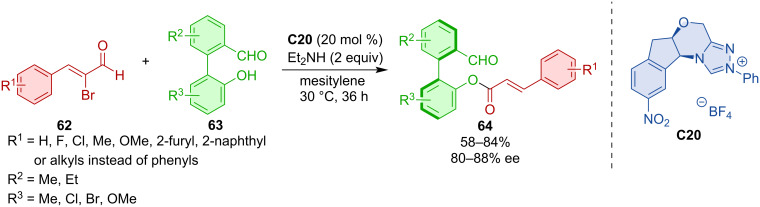
NHC-catalyzed DKR of 2-arylbenzaldehydes **62**.

The dynamic kinetic resolution (DKR) of racemic 2-arylbenzaldehydes **62** with α-bromoenals **63** led to axially chiral products **64** [43]. Triazolium salt **C20** as an NHC pre-catalyst was the most efficient for achieving high yields and enantiomeric purities in this process (Scheme 20).

### Chiral Brønsted acid-catalyzed atroposelective reactions

**Scheme 21:**
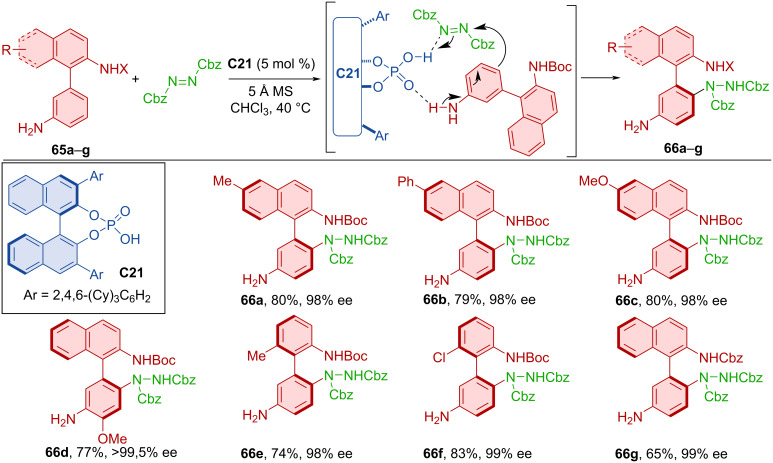
Atroposelective biaryl amination.

Chiral Brønsted acids became prominent organocatalysts that also promote the syntheses of axially chiral compounds. The amination of aromatic biaryls **65a**–**g** with dibenzylazodicarboxylate catalyzed by organocatalyst **C21** was studied in 2019 (Scheme 21) [44]. A broad range of aniline and phenol substrates was studied. The best results were accomplished with products containing a Boc-protected amino group on the aniline or 2-aminonaphthalene frame (**66a**–**g**), achieving very good yields and excellent enantioselectivities. Compound **66d** was incorporated into a thiourea organocatalyst framework and successfully tested in kinetic resolution with 73% enantioselectivity.

**Scheme 22:**
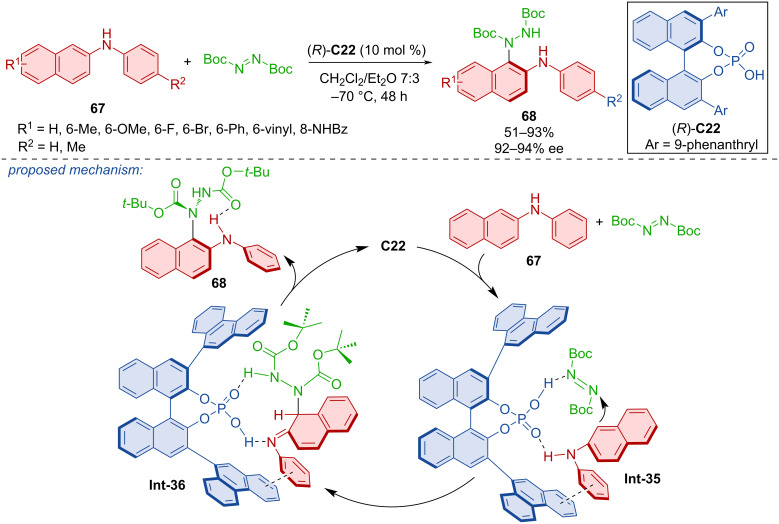
CPA-catalyzed atroposelective amination of 2-anilinonaphthalenes.

The chiral phosphoric acid (CPA) (*R*)-**C22** was used to catalyze the formation of a C–N chiral axis in the axially chiral product **68** from biarylamines **67** and di-*tert*-butyl azodicarboxylate (Scheme 22) [45]. An added benefit to these products is that they possess an intramolecular hydrogen bond acting as a stabilizing factor and products **68** were prepared in good to very good yields and excellent enantiomeric purities. Based on the authors’ design, previous findings from the literature, and experimental results, a reaction mechanism was proposed [46]. Hydrogen bonding as well as π–π interaction with the catalyst (*R*)-**C22** activates both substrates in the stable intermediate **Int-35**. This stabilized state ensures the concerted control of enantioselectivity during the nucleophilic addition, and the subsequent aromatization completes central-to-axial chirality conversion delivering products **68**.

**Scheme 23:**
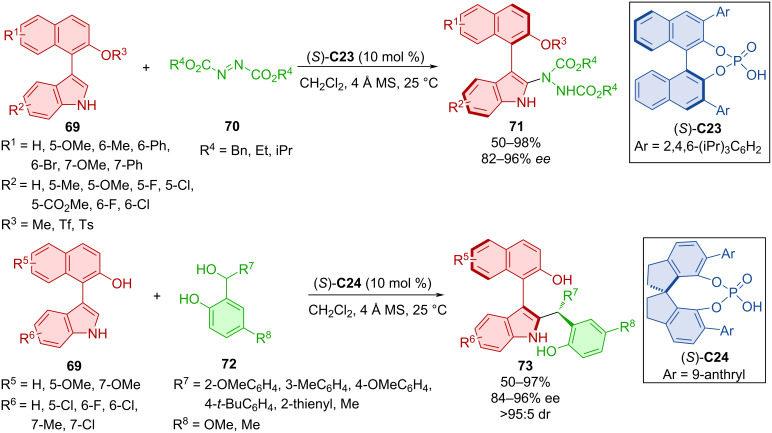
Atroposelective DKR of naphthylindoles.

Dynamic kinetic resolution of naphthylindoles **69** was performed by reaction with bulky electrophiles such as azodicarboxylates **70** or *o*-hydroxybenzyl alcohols **72** (Scheme 23) [47]. These reactions were catalyzed by both BINOL-derived (TRIP) CPA (*S*)-**C23** and SPINOL-derived CPA (*S*)-**C24**, providing axially chiral products **71** and **73**, respectively. Control experiments showed the importance of the N–H group on the indole ring and the presence of both carboxylate groups in the azodicarboxylate as crucial to forming hydrogen bonds with the organocatalyst. Benzylation of this nitrogen or substitution of just one of the carboxylate groups led to no product being observed. A series of naphthylindoles **71** was tested for potential biological activity and showed promising results in one case, providing high cytotoxicity toward the MCF-7 cancer cell line. The stable axial chirality of the products **71** and **73** was confirmed by calculations of the rotational barriers ranging from 30.2 to 46.3 kcal/mol.

**Scheme 24:**
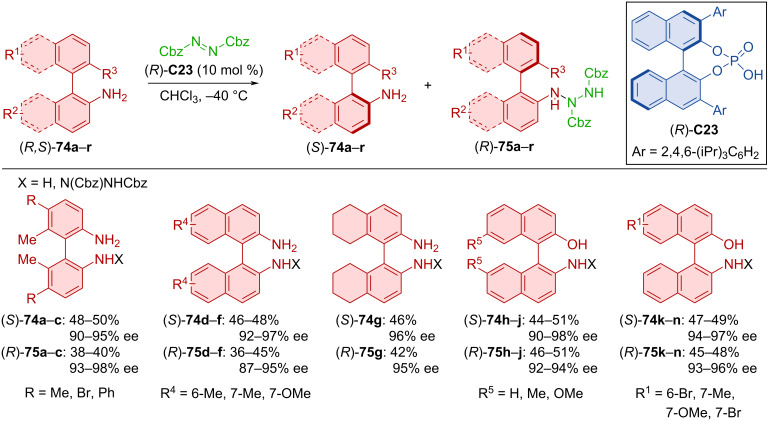
CPA-catalyzed kinetic resolution of binaphthylamines.

Kinetic resolution by amino group protection of biaryls (*R,S*)-**74a**–**r** with azodicarboxylate catalyzed by CPA (*R*)-**C23** provided axially chiral unprotected biaryls (*S*)-**74a**–**r** and axially chiral protected biaryls (*R*)-**75a**–**r** (Scheme 24) [48]. Consistently high enantioselectivities and yields were reported with various binaphthyl and biphenyl substrates. Control experiments revealed the importance of hydrogen on the amino or hydroxy groups, supposedly through the bonding with the catalyst. Substitution of these groups or their hydrogens led to either halted reaction or significantly reduced enantiopurity of the products.

**Scheme 25:**
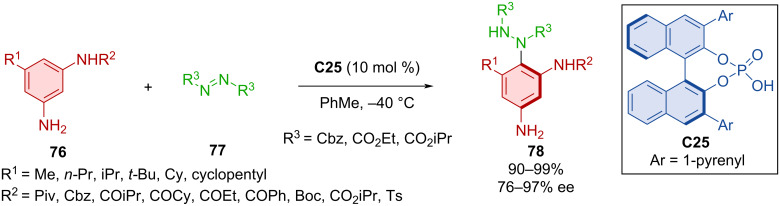
Atroposelective amination of aromatic amines with diazodicarboxylates.

Expanding the scope of available azodicarboxylates **77** and aromatic amines **76** in the C–H amination reaction with CPA **C25**, the authors were able to prepare axially chiral *para*-amination products **78** (Scheme 25) [49]. Such amination products **78** were prepared with high levels of yields and showed remarkable enantiomeric purities. Interestingly, when a phenyl substituent was present on the amino group of the 1,3-benzenediamine, lower yields were reported, and substituting the amino group in position 3 for an *N*-methylamino or *N,N*-dimethylamino group led to a reduction in the enantioselectivity.

**Scheme 26:**
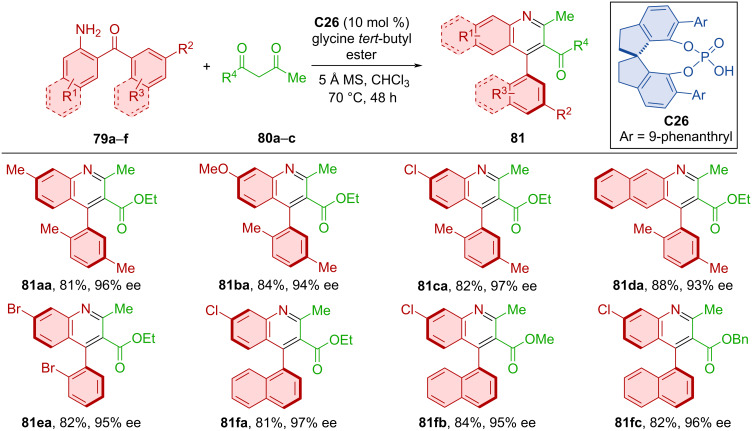
Atroposelective Friedländer heteroannulation.

Shao et al. developed the first organocatalyzed atroposelective Friedländer heteroannulation [50]. The SPINOL-derived chiral phosphoric acid **C26** catalyzed the formation of axially chiral products **81** from diarylketones **79a**–**f** and ketoesters **80a**–**c** (Scheme 26). The substrate scope contained a broad range of substituents, including electron-donor groups and whole benzene rings. The authors were able to separate the enamine intermediate formed from diarylketone and ketoester.

**Scheme 27:**
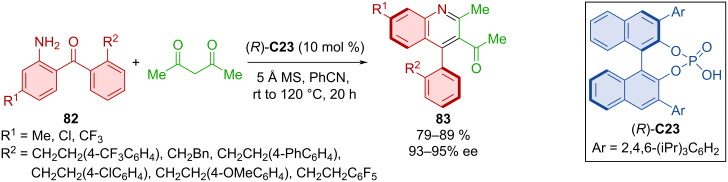
CPA-catalyzed formation of axially chiral 4-arylquinolines.

Later, the BINOL-derived (TRIP) CPA (*R*)-**C23** was used in a similar Friedländer reaction [51]. Acetylacetone was utilized with diarylketones **82** containing arylethyl chains to form axially chiral products **83** (Scheme 27). The reaction mechanism proposed by the authors was analogous to that of the aforementioned atroposelective Friedländer reaction. Outstanding yields and enantioselectivities were accomplished during the substrate scope screening as well as in a model gram-scale reaction (83%, 91% ee).

**Scheme 28:**
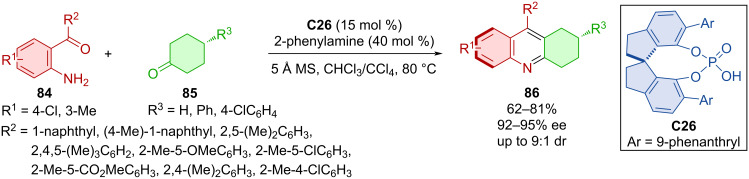
CPA-catalyzed Friedländer reaction of arylketones with cyclohexanones.

Annulation of biaryl ketones **84** with cyclohexanones **85** mediated by the second-generation chiral phosphoric acid **C26** led to the formation of tetrahydroacridines **86** (Scheme 28) [52]. This Friedländer reaction provided products **86** in moderate to good yields with consistently high enantiomeric purities and high diastereomeric ratios in a couple of cases. On a 1 mmol scale with reduced catalyst loading the reaction proceeded in a similar fashion with good yield and enantioselectivity (70%, 89% ee).

**Scheme 29:**
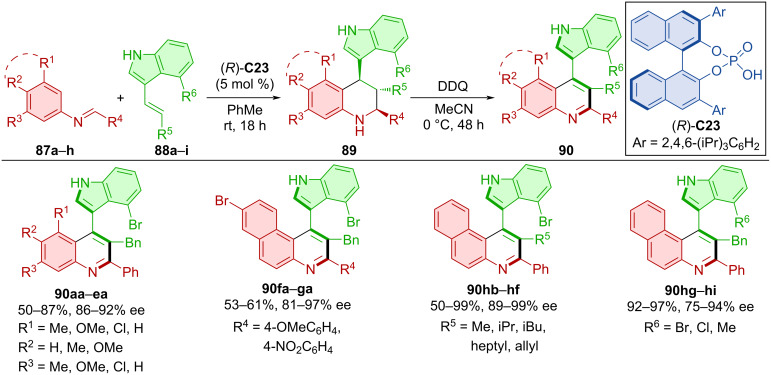
CPA-catalyzed atroposelective Povarov reaction.

The Povarov reaction of imines **87a**–**h** and alkenylindoles **88a**–**i** catalyzed by CPA (*R*)-**C23** was utilized to give asymmetric products **89** and their subsequent oxidation with DDQ provided axially chiral quinolines **90** (Scheme 29) [53]. Good retention of the stereoinformation acquired in the first transformation, moderate to excellent yields and consistently high degrees of enantiomeric purity were achieved. The reaction could also be carried out in a one-pot fashion with comparable results and without significant variation from the two-step procedure.

**Scheme 30:**
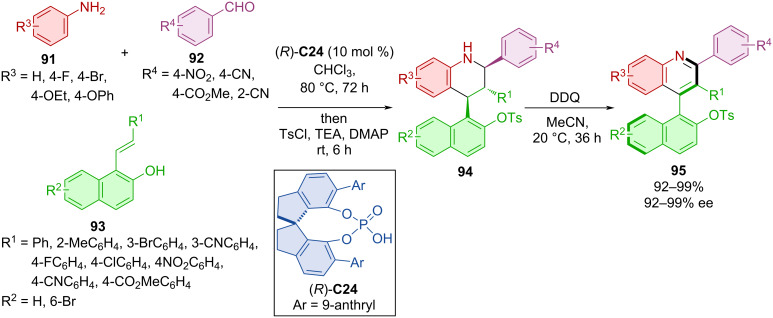
Atroposelective CPA-catalyzed Povarov reaction.

Utilization of the Povarov reaction and subsequent oxidation by DDQ was also done by Wang et al. in 2020 [54]. In situ-formed imines from anilines **91** and benzaldehydes **92** were reacted with alkenyl-2-naphthols **93** in the presence of CPA (*R*)-**C24** to form asymmetric products **94** and eventually axially chiral tetrahydroquinolines **95** through oxidation (Scheme 30). This approach led to the products **95** in high yields and enantiomeric purities. The tosyl group in the product was transformed through a series of reactions to a diphenylphosphine group and used as a ligand for Pd-catalyzed reactions.

**Scheme 31:**
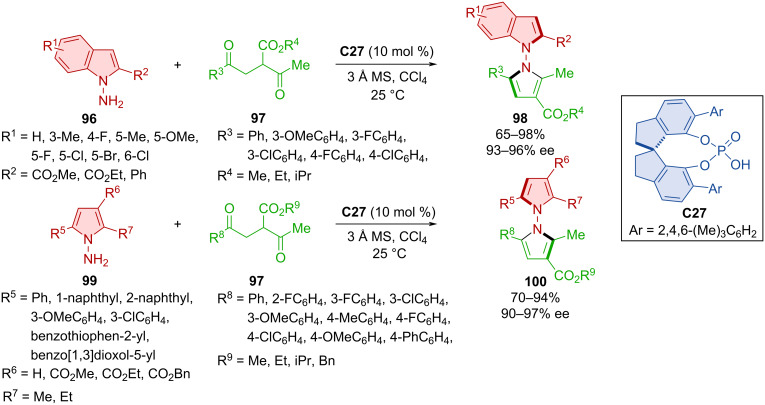
Paal–Knorr formation of axially chiral *N*-pyrrolylindoles and *N*-pyrrolylpyrroles.

De novo ring formation was utilized in the synthesis of N–N axially chiral *N*-pyrrolylindoles **98** and *N*-pyrrolylpyrroles **100** with the help of CPA **C27** (Scheme 31) [55]. Starting from either indoles **96** or pyrroles **99** and 1,4 diketones **97**, respectively, the authors were able to achieve very good to near-perfect yields with consistently high enantioselectivities. Configurational stabilities of the products **98** and **100** were explored in toluene at 110 °C. Rotational barriers were calculated to be 47.7 and 52.2 kcal/mol, respectively, which suggests a high degree of configurational stability. One-mmol-scale reactions provided the corresponding products in comparable yields and enantioselectivities (87–96%, 94–97% ee). Based on a previous report on the CPA-catalyzed Paal–Knorr reaction, a reaction pathway was proposed [56]. The first step is a CPA **C27**-catalyzed condensation giving rise to the imine intermediate followed by isomerization to the enamine stabilized by CPA. An enantioselective intramolecular cyclization followed by dehydration then afford the aromatic ring and desired product **98**.

**Scheme 32:**
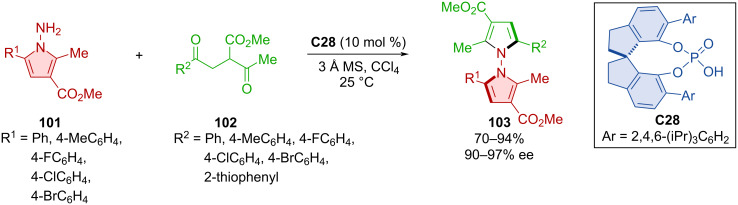
Atroposelective Paal–Knorr reaction leading to *N*-pyrrolylpyrroles.

Concurrently, Gao et al. utilized a similar Paal–Knorr reaction for the synthesis of axially chiral biheteroaryls **103** (Scheme 32) [57]. In the majority of the experiments Fe(OTf)_3_ was utilized as Lewis acid. The below-mentioned examples are only those, that did not require an additional co-catalyst containing transition metals but are purely of organocatalytic nature. In these experiments, the chiral phosphoric acid **C28** catalyzed the reaction of aminopyrroles **101** and 1,4-diketones **102**. Under the optimized reaction conditions, the yields were good to excellent, and high levels of enantioselectivities were achieved. The products showed no thermal racemization at 150 °C, what was supported by a calculated high rotational barrier of 49.9 kcal/mol.

**Scheme 33:**
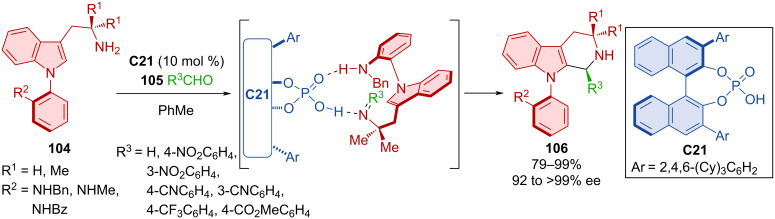
Atroposelective Pictet–Spengler reaction of *N*-arylindoles with aldehydes.

The usefulness of chiral phosphoric acids also shows in the atroposelective Pictet–Spengler reaction of *N*-arylindoles **104** with various aldehydes **105** (Scheme 33) [58]. Axially chiral products **106** were prepared in very high yields and exquisite enantiomeric purities. The presence of a methyl group in the aniline ring's *ortho* position proved to have a negative effect on the enantioselectivity, presumably due to the unfavorable steric interaction with the organocatalyst **C21**. A considerable drop in yield and enantioselectivity was also observed in the reaction with dibenzylaniline. Interactions with and steric effects of the CPA **C21** guiding the orientation of the substrates dictate the stereocontrol of the reaction.

**Scheme 34:**
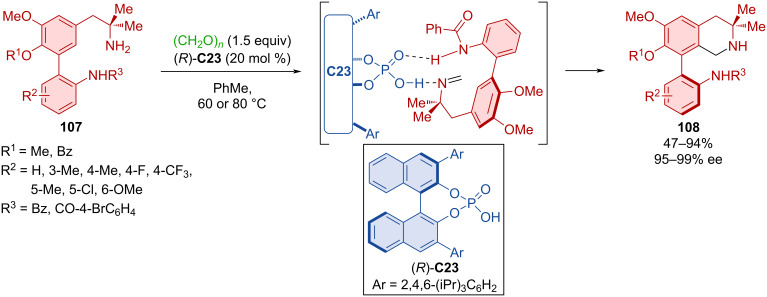
Atroposelective Pictet–Spengler reaction leading to tetrahydroisoquinolin-8-ylanilines.

The utilization of CPA (*R*)-**C23** in a dynamic kinetic resolution through a Pictet–Spengler reaction, enabled the preparation of axially chiral 8-aryltetrahydroisoquinolines **108** starting from aminobiaryl scaffolds **107** and paraformaldehyde (Scheme 34) [59]. For most substrates, excellent enantioselectivities and moderate to excellent yields were reported. However, the reaction did not tolerate a variety of substitutions on the amide group, probably because of its involvement in hydrogen bonding with the organocatalyst (*R*)-**C23**.

**Scheme 35:**
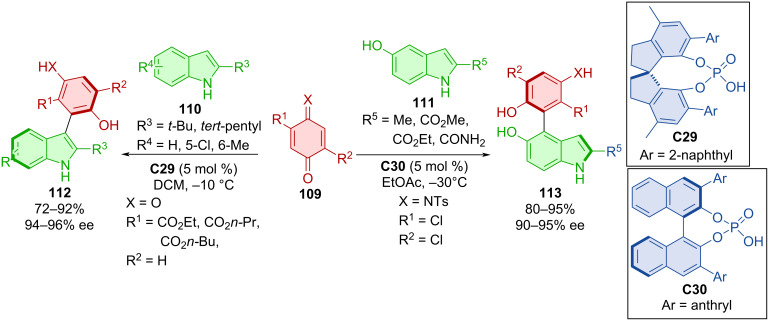
Atroposelective formation of arylindoles.

Expanding on earlier methodologies of Chen et al. [60–61] and Wang et al. [62] utilizing indole derivatives instead of β-naphthols, new atroposelective reactions of quinones and iminoquinones were developed [63]. The reaction of quinones with an ester group **109** and indoles with alkyl substituents **110** catalyzed by CPA **C29** provided products **112** with regioselectivity on the pyrrole ring of indole (Scheme 35). On the contrary, adding a hydroxy group to the benzene ring of indoles **111** and reacting them with tosyl-protected iminoquinone **109** with the help of CPA **C30** led to the shift in regioselectivity providing different axially chiral products **113**. All products were obtained with high degree of enantiomeric purity as well as significantly high yields. The CPA organocatalyst activates quinones with an acceptor hydrogen bond while indole acts as hydrogen-bond donor. On the other hand, a hydroxy group of hydroxyindole becomes a hydrogen donor and the iminoquinone nitrogen represents an acceptor to the hydrogen from the CPA, resulting in the regioselectivity change.

**Scheme 36:**
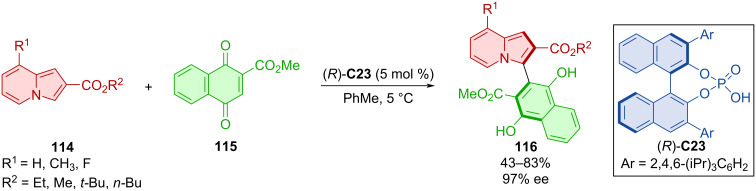
CPA-catalyzed arylation of naphthoquinones with indolizines.

Song et al. broadened the scope of usable substrates for the asymmetric arylation of naphthoquinones **115** with indolizines **114** catalyzed by CPA (*R*)-**C23** forming atropoisomers **116** (Scheme 36) [64]. A broad range of indolizine substrates was tested with substituents in positions 6 and 7, but the best results were consistently achieved by exchanging the hydrogen in position 8. Such products along with the ones containing various butyl, methyl, and ethyl esters on the indolizine ring were obtained in moderate to good yields with repeatedly high enantiomeric purity of 97%.

**Scheme 37:**
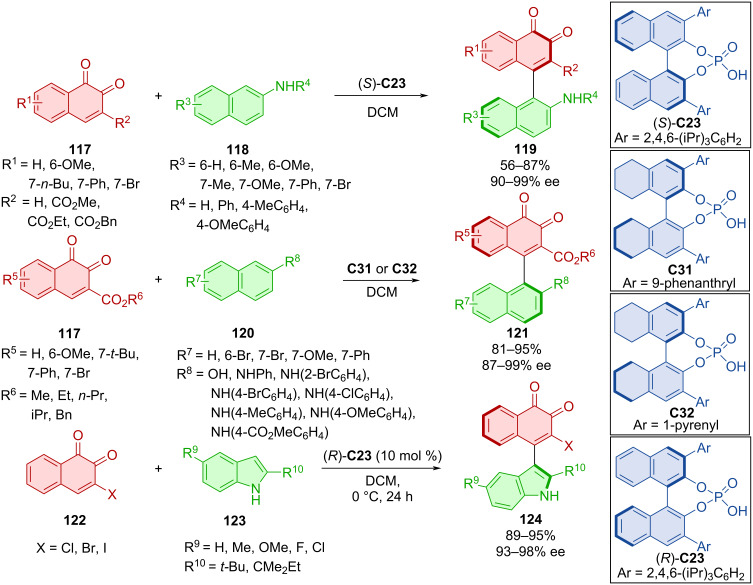
Atroposelective reaction of *o*-naphthoquinones.

An extensive study on the reactivity of *o*-naphthoquinones **117** and **122** with 2-naphthylamines, 2-naphthols (**118**, **120**), and indoles **123** was done in 2019 (Scheme 37) [65]. Four organocatalysts ((*S*)-**C23**, **C31**, **C32**, (*R*)-**C23**) proved the most efficient, and stereoinformation was effectively transferred in all cases. High yields and remarkable enantiomeric purities were achieved with all prepared products (**119**, **121**, and **124**).

The proposed reaction pathway indicates the asymmetric conjugated addition from 2-naphthylamine, stabilized by a donor hydrogen bond to the organocatalyst, towards the *o*-naphthoquinone, stabilized with an acceptor hydrogen bond to the chiral phosphoric acid.

**Scheme 38:**
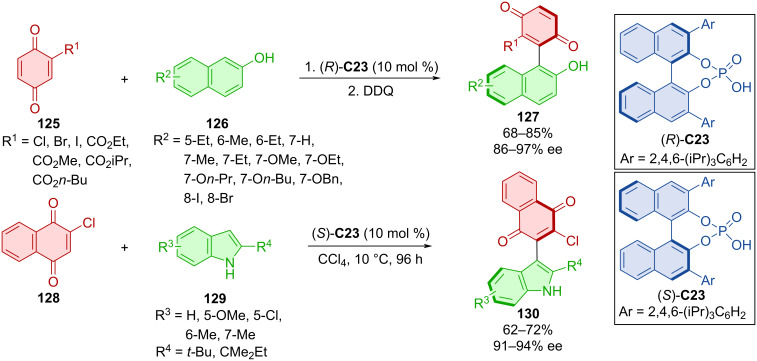
CPA-catalyzed formation of axially chiral arylquinones.

Chen et al. developed an organocatalytic atroposelective preparation of arylquinones **127** and **130** utilizing CPA enantiomers (*R*)-**C23** and (*S*)-**C23** (Scheme 38) [66]. In one case quinones **125** were reacted with 2-naphthols **126** and after subsequent oxidation with DDQ provided the respective products **127**. When indoles **129** were utilized with quinone **128**, no further oxidizing reagents were necessary to afford indolylquinones **130**. All products were obtained with high enantiomeric ratios and moderate to good yields. A model reaction performed at a gram scale gave the product with analogous yield and enantioselectivity (73%, 96% ee).

**Scheme 39:**
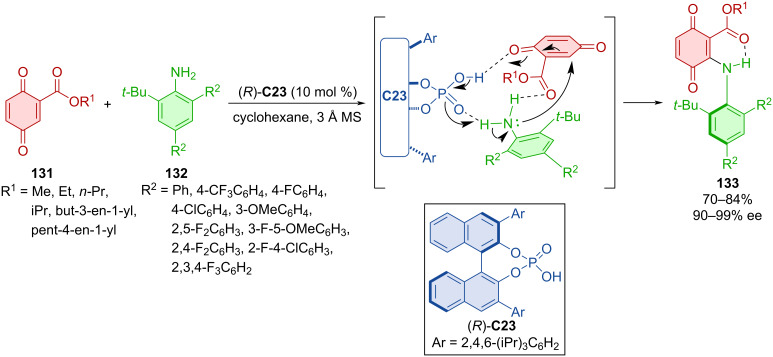
CPA-catalyzed axially chiral *N*-arylquinones.

Hydrogen-bond-stabilized axially chiral *N*-arylquinones **133** were prepared by reaction of quinone esters **131** with anilines **132** mediated by CPA (*R*)-**C23** (Scheme 39) [67]. Apart from respectable yields and remarkable enantioselectivities, the authors also calculated the racemization barrier of products **133**. It was determined as a class-3 atropoisomer with 30.1 kcal/mol at 90 °C in toluene. The preparation of **133** on a gram scale yielded 75% of the product with a similar level of enantiomeric purity (90% ee). A key step of this transformation is an asymmetric conjugate addition leading to a central chiral intermediate that tautomerizes to the axially chiral product.

**Scheme 40:**
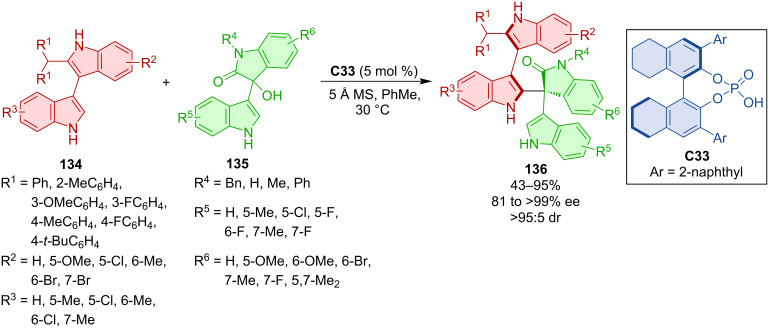
Atroposelective additions of bisindoles to isatin-based 3-indolylmethanols.

Chiral phosphoric acid **C33** was utilized in the construction of products **136** bearing both axial and central chirality (Scheme 40) [68] through the reaction of bisindoles **134** and isatin-derived 3-indolylmethanols **135**. Over 90% diastereoselectivity, mostly very good yields, and consistently high enantioselectivities were reported. Testing the practicality of the protocol, the gram-scale experiment provided representative product **136** in 93% yield and an excellent stereoselectivity (96% ee, >95:5 dr). No racemization was observed after 36 hours at 150 °C in *o*-xylene.

**Scheme 41:**
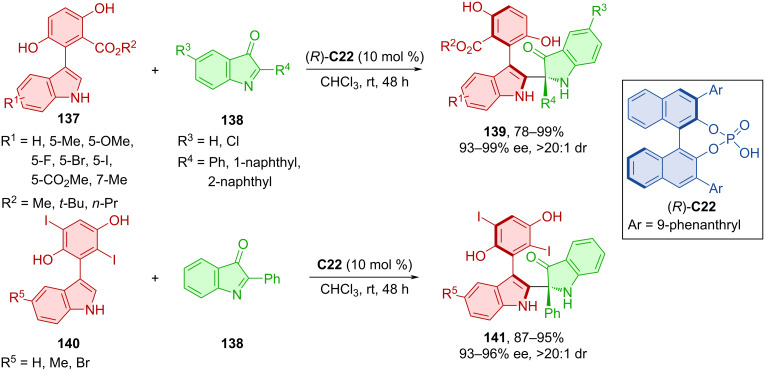
CPA-catalyzed synthesis of axially chiral arylindolylindolinones.

The asymmetric synthesis of arylindolylindolinones **139** or **141** bearing both central and axial chirality was accomplished by a combination of arylindoles **137** or **140** and indolinones **138** with CPA (*R*)-**C22** acting as the catalyst (Scheme 41) [69]. Following identical reaction conditions two series of different atropoisomers were prepared with very high yields, enantioselectivities, and diastereoselectivities. The corresponding products proved to be stable for up to 12 h in *o*-xylene at 120 °C. Methylation of the indole nitrogen in control experiments led to halted reactivity or loss of enantiocontrol. These results suggest the importance of hydrogen bonding between the NH group and the organocatalyst.

**Scheme 42:**
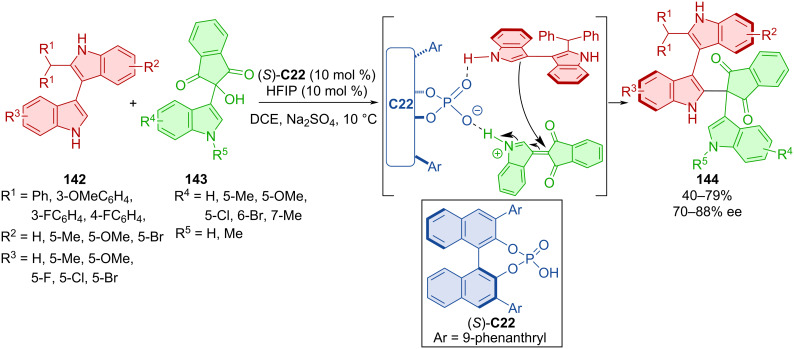
CPA-catalyzed reaction between bisindoles and ninhydrin-derived 3-indoylmethanols.

Bisindoles **142** reacted with ninhydrin-derived 3-indoylmethanol **143** in the presence of the CPA (*S*)-**C22** to afford axially chiral products **144** (Scheme 42) [70]. Moderate to decent yields were reported, with enantioselectivities up to 88%. In terms of configurational stability, the representative product **144** could be stirred at 130 °C for 24 h in toluene without loss in yield or enantioselectivity. The calculated rotational barrier of the same compound was found to be 43.8 kcal/mol, which is higher by approximately 30.7 kcal/mol than that of the starting material. This value classifies the corresponding product **144** as class-3 atropoisomer [4]. Experimental results helped with providing possible reaction pathways. The acidic hydrogen of the CPA promotes dehydration and the formation of the vinyliminium intermediate. Chirality control is consistent because of the retarded reaction with the unfavored enantiomer of the bisindole **142** and its low rotational barrier resulting in quick exchange between the two.

**Scheme 43:**
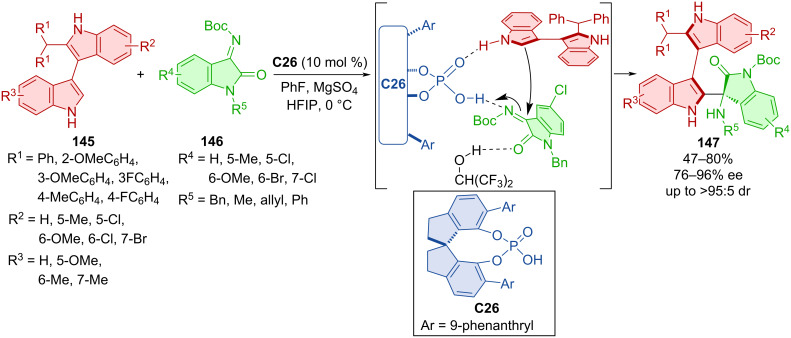
Atroposelective reaction of bisindoles and isatin-derived imines.

Products **147**, bearing both axial and central chirality, were prepared by organocatalytic asymmetric addition of bisindoles **145** and isatin-derived imines **146** catalyzed by CPA **C26** (Scheme 43) [71]. The scope of the reaction showed efficient stereocontrol by consistently high diastereo- and enantioselectivity with moderate to high yields. A one-mmol-scale reaction of the corresponding product showed a higher yield and similar enantioselectivity. Compared to the low rotational barrier of bisindoles (13.1 kcal/mol), a much higher value (46.3 kcal/mol) was calculated for the final product of the respective reaction. Experiments determining the configurational stability were done in both toluene at 120 °C and *o*-xylene at 150 °C for up to 36 h with retained stereoselectivity but decreasing yields at higher temperatures for prolonged periods of time. The possible activation mode explains the stereocontrol of the reaction with key hydrogen bonds between substrates, organocatalyst, and HFIP.

**Scheme 44:**
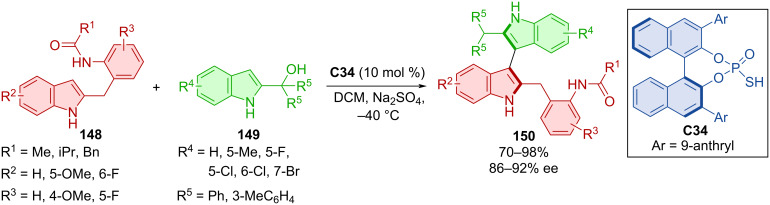
CPA-catalyzed formation of axially chiral bisindoles.

Sheng et al. utilized the BINOL-derived organocatalyst **C34** in the reaction of benzylindoles **148** and 2-indolylmethanols **149** leading to the bisindoles **150** (Scheme 44) [72]. Very good yields and good to high enantioselectivities were reported. The configurational stability and rotational barrier were also investigated. The enantioselectivity gradually decreased at 70 °C for 12 h in isopropyl alcohol and the calculated value was 28.5 kcal/mol. Control experiments proved the importance of N–H bonds during the reaction and methylation of just one nitrogen resulted in retarded or halted reactivity.

**Scheme 45:**
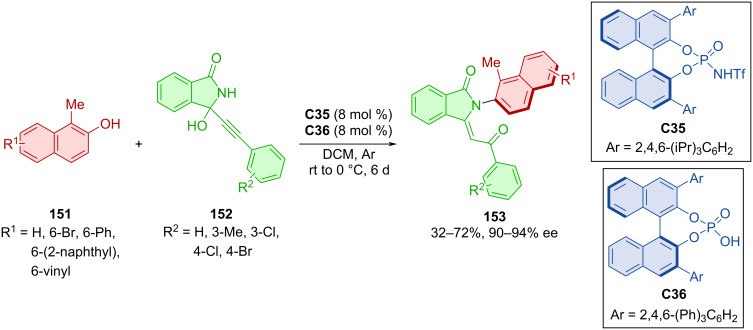
Atroposelective reaction of 2-naphthols with alkynylhydroxyisoindolinones.

The combination of 2-naphthols **151** and alkynylhydroxyisoindolinones **152** in the presence of two chiral Brønsted acids **C35** and **C36** provided axially chiral isoindolinones **153** (Scheme 45) [73]. The optimized reaction conditions led to the handful of products in low to satisfactory yields, but having high enantiomeric purities.

**Scheme 46:**
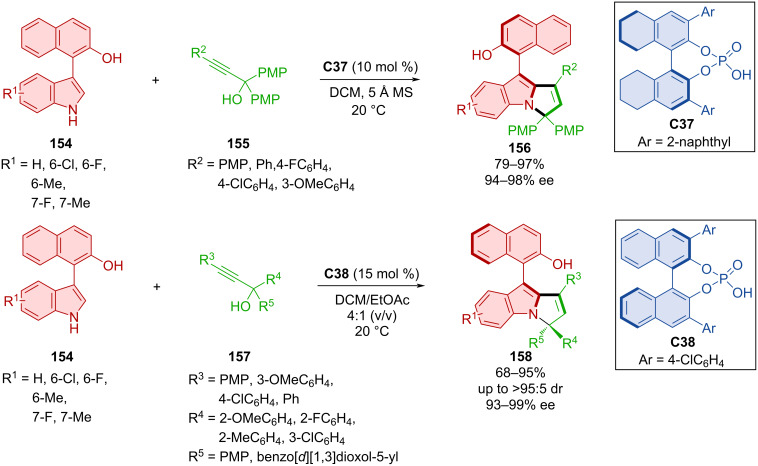
CPA-catalyzed reaction of indolylnaphthols with propargylic alcohols.

An organocatalytic asymmetric (3 + 2) cyclization of 3-arylindoles **154** with either achiral **155** or racemic **157** propargylic alcohols was reported by Wu et al. (Scheme 46) [74]. Utilizing CPA **C37** with 3-arylindoles **154** and achiral propargylic alcohols **155**, the axially chiral arylpyrrolindoles **156** were prepared with excellent enantiomeric purities and high yields. On the other hand, using racemic propargylic alcohols **157** and same 3-arylindoles **155** with CPA **C38** led to the opposite enantiomers **158** with remarkable enantiomeric purities, solid yields and very good diastereomeric ratios. The hydroxy group present in products **156** and **158** could be transformed to provide axially chiral phosphines that could be utilized as chiral ligands in transition-metal-catalyzed reactions. Testing both substrates **156** and **158** for conformational stability in isopropanol at 80 °C for 12 h provided recovered substrates in high yields with maintained diastereo- and enantioselectivities. Based on calculations of rotational barriers, these compounds meet the requirements to be considered class-3 atropoisomers (32.9–37.7 kcal/mol).

**Scheme 47:**
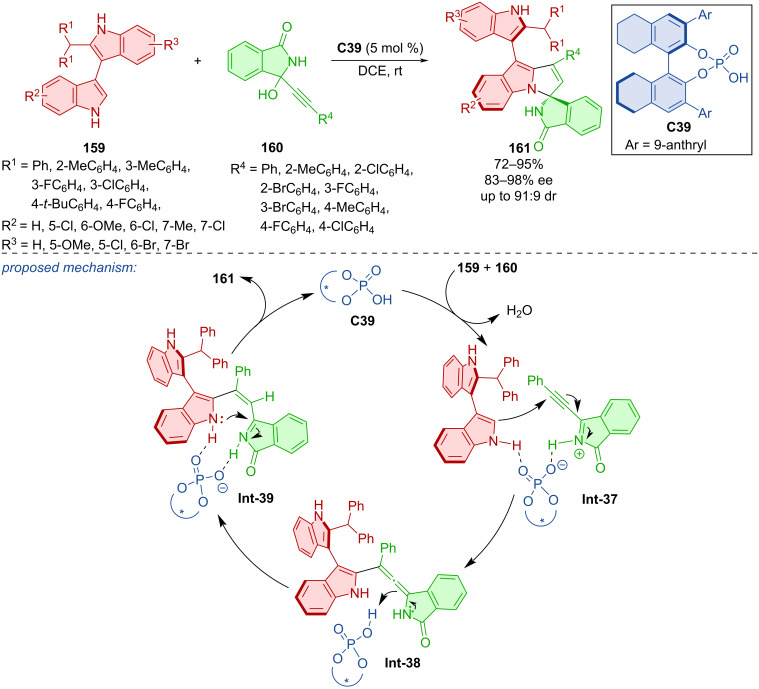
Atroposelective formation of indolylpyrroloindoles.

Indolylpyrroloindoles **161** were constructed by a (3 + 2) cycloaddition of isoindolinone-substitued propargylic alcohols **160** with bisindoles **159** mediated by CPA **C39** (Scheme 47) [75]. These axially chiral spirocyclic products were prepared with good to very good yields and excellent enantiomeric purities. The slow racemization process of **161** was observed at 70 °C, and the experimentally determined rotational barrier of 30.5 kcal/mol was observed at 100 °C in toluene. Control experiments gave insights into the potential importance of hydrogen bonds on the nitrogen atoms of the isoindolinone ring in **160** and one indole ring in **159**. Based on these findings, a potential reaction pathway was proposed. It starts with a chiral phosphoric acid-supported dehydration of **160** and reaction with the favored configuration of bisindoles **159** to form an allene intermediate **Int-38**. Proton transfer and subsequent intramolecular cycloaddition occurs to generate indolylpyrroloindole **161**.

**Scheme 48:**
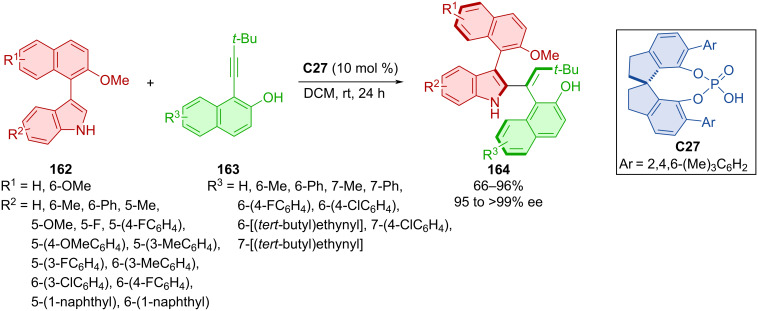
Atroposelective reaction of indolylnaphthalenes with alkynylnaphthols.

Woldegiorgis et al. developed an efficient atroposelective synthesis of axially chiral styrenes connected to axially chiral naphthylindoles **164** from naphthylindoles **162** and alkynyl-2-naphthols **163** catalyzed by CPA **C27** (Scheme 48) [76]. The reaction conditions were compatible with many substrates containing methyl, methoxy, halogen, and aryl groups, providing excellent enantioselectivies and moderate to high yields. Control experiments indicated an activation mode through the vinylidene *ortho*-quinone methide (VQM) intermediate as well as the importance of the naphthol's OH group and indole's NH group, presumably through hydrogen bonding with organocatalyst **C27**.

**Scheme 49:**
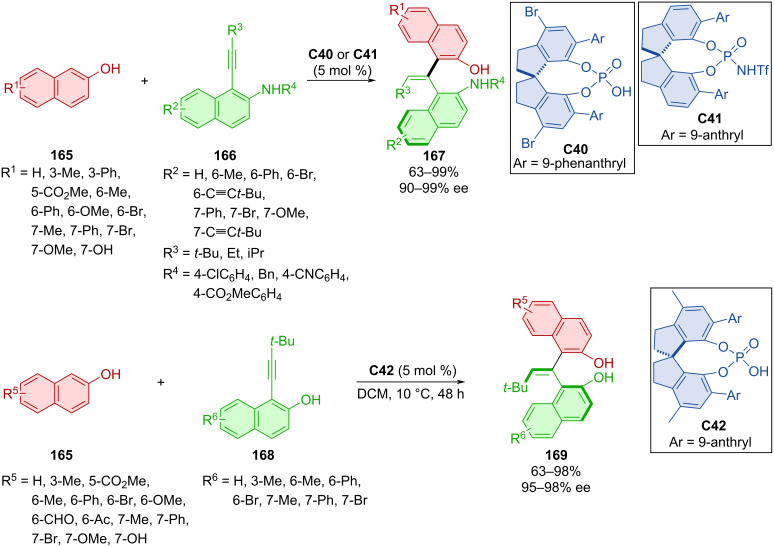
CPA-catalyzed addition of naphthols to alkynyl-2-naphthols and 2-naphthylamines.

The organocatalytic atroposelective preparation of promising EBINOL scaffolds **167** and **169** was done by Wang et al. with the help of the SPINOL-derived organocatalysts **C40**, **C41**, and **C42** (Scheme 49) [77]. They reacted 2-naphthols **165** either with alkynyl-2-naphthylamines **166** or alkynyl-2-naphthols **168**, respectively. Decent results were achieved with structures containing electron-withdrawing groups such as methyl carboxylate, acetyl, and formyl. The prepared EBINOL **169** was transformed into a CPA to be used as an organocatalyst or to a phosphoramidite to be used as a chiral ligand. Testing these new structures on known stereoselective transformations, the authors achieved high yields and enantioselectivities (up to 98% yield and 97% ee).

**Scheme 50:**
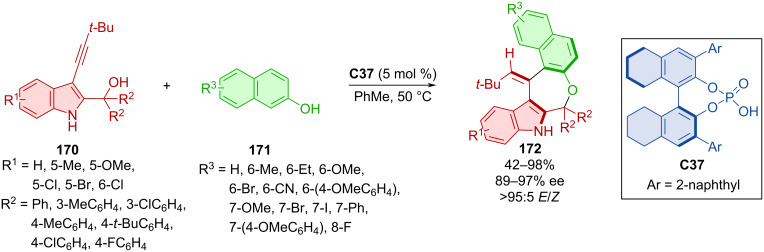
CPA-catalyzed formation of axially chiral aryl-alkene-indoles.

Wang et al. performed asymmetric (4 + 3) cyclization of alkynylindolylmethanols **170** and 2-naphthols **171** mediated by chiral phosphoric acid **C37** leading to axially chiral aryl-alkene-indoles **172** (Scheme 50) [78]. Very high enantioselectivities and *E*/*Z* ratios, along with, on average, decent yields, were reported. Slow racemization was observed at 40 or 50 °C in isopropanol just after a couple of hours. The racemization barriers of the products were only slightly higher (28 kcal/mol) than the minimal requirement for separation of atropoisomers (24 kcal/mol). Scale-up done on the one-mmol-scale provided the corresponding product in comparable yields and stereoselectivities (85%, 90% ee, >95:5 *E/Z*). A biological activity investigation led to promising results in the case of one substrate displaying cytotoxicity towards several cancer cell lines.

**Scheme 51:**
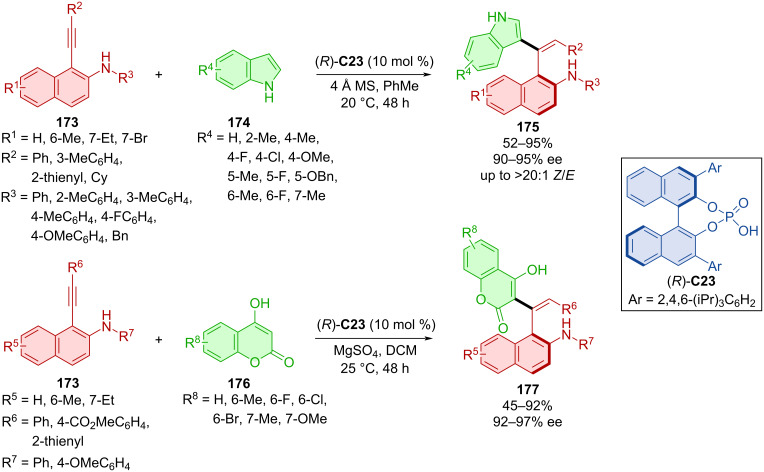
CPA-catalyzed formation of axially chiral styrenes.

Organocatalytic enantioselective construction of axially chiral styrenes **175** and **177** was done utilizing alkynylnaphthylamines **173** with indoles **174** or coumarins **176** catalyzed with CPA (*R*)-**C23** (Scheme 51) [79]. The extensive scope of alkynylnaphthylamines **173** and indoles **174** led to the products **175** in moderate to high yields with very high enantiomeric purities and decent *E*/*Z* ratio. A gram-scale verson of the reaction provided the product with comparable yield and enantiomeric purity (88%, 93% ee). The more modest scope of coumarins **176** with alkynylnaphthylamines **173** gave rise to axially chiral products **177** in comparable yields to the first reaction with slightly higher enantiomeric purities in general.

**Scheme 52:**
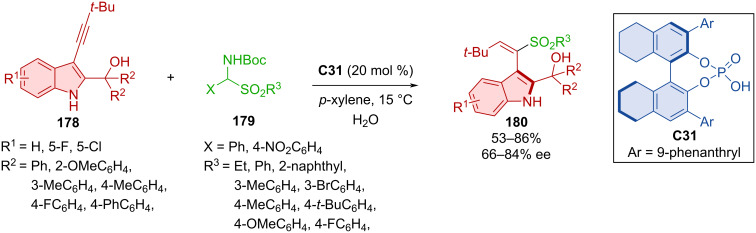
Atroposelective formation of alkenylindoles.

Axially chiral alkenylindoles **180** were prepared by the addition of α-amido sulfones **179** to triple bond of alkynylindolylmethanols **178** catalyzed by CPA **C31** (Scheme 52) [80]. Authors achieved mild to good yields with decent to very good enantioselectivities. Alkenylindole **180** was used for the thermal racemization experiment, which revealed a rotational barrier of 43.2 kcal/mol.

**Scheme 53:**
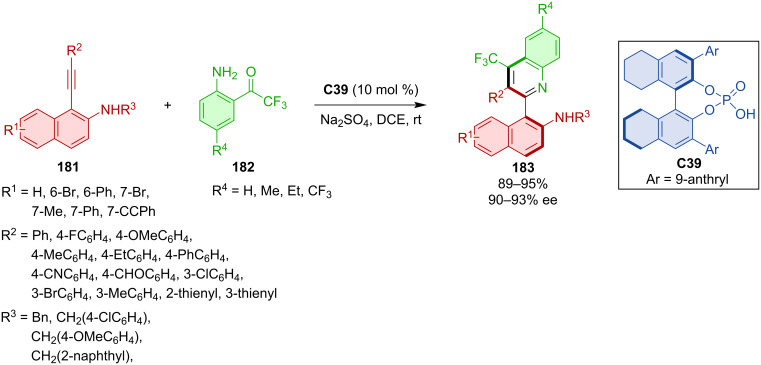
Atroposelective formation of axially chiral arylquinolines.

Axially chiral arylquinolines **183** were prepared starting from alkynylnaphthylamines **181** and acetylanilines **182** with the help of CPA **C39** (Scheme 53) [81]. Excellent yields and enantioselectivities were reported for a plenty of different derivatives. The proposed reaction pathway follows hydrogen bonding with alkynylnaphthylamine and later nucleophilic addition of the allene intermediate. The synthesis on the preparative scale provided product **183** with almost no deterioration in yield or enantioselectivity (90%, 91% ee). This product could then be either debenzylated and subsequently transformed into a thiourea organocatalyst or turned into an axially chiral sulfonamide.

**Scheme 54:**
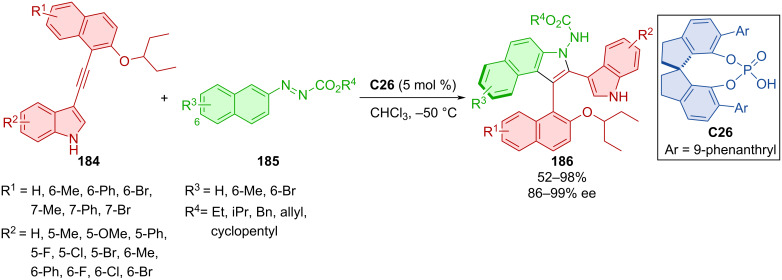
Atroposelective (3 + 2) cycloaddition of alkynylindoles with azonaphthalenes.

In 2022, Yang et al. presented their (3 + 2) formal cycloaddition of alkynylindoles **184** with azonaphthalenes **185** catalyzed by the SPINOL-based CPA **C26** (Scheme 54) [82]. The authors prepared a wide scope of axially chiral products **186** in high yields with excellent enantiomeric purity. The reaction allows lowering of the catalyst loading to 2 mol %. Deprotection of the amino group enabled subsequent transformations, such as a reaction with isocyanate from which a new potential thiourea organocatalyst was prepared.

**Scheme 55:**
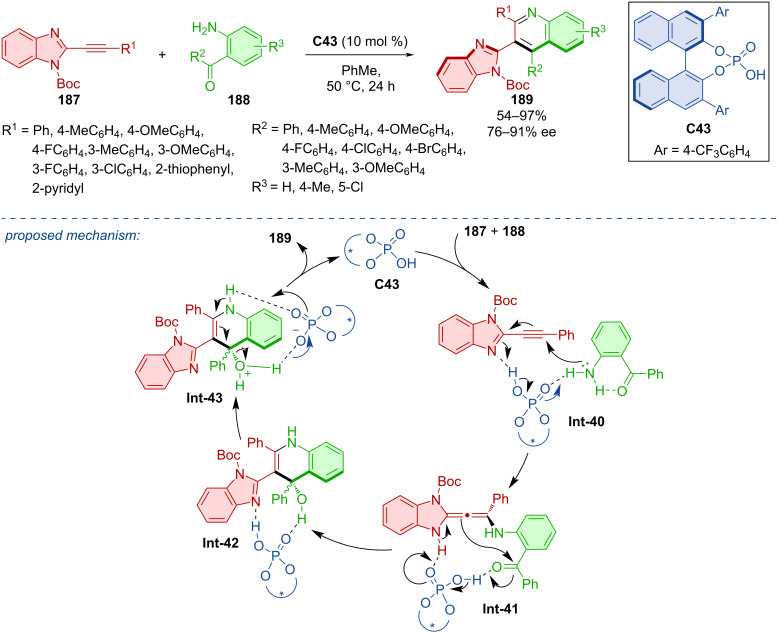
CPA-catalyzed formation of axially chiral 3-(1*H*-benzo[*d*]imidazol-2-yl)quinolines.

Hou et al. investigated a way to prepare axially chiral compounds that contain both benzimidazole and quinoline rings **189** (Scheme 55) [83]. One route to access such compounds was possible through the reaction of 2-alkynylbenzimidazoles **187** with *o*-aminophenylketones **188** mediated by the chiral phosphoric acid **C43**. In general, moderate to excellent yields and very good enantioselectivities were reported. The authors presented a reaction mechanism based on experimental outcomes and previous reports [84–85]. The reaction of the substrates stabilized by hydrogen bonding with catalyst **C43** leads to allene intermediate **Int-41**. The enantiospecific intramolecular enamine–aldol cyclization and further dehydration provide the enantioenriched heterobiaryl product **189**.

**Scheme 56:**
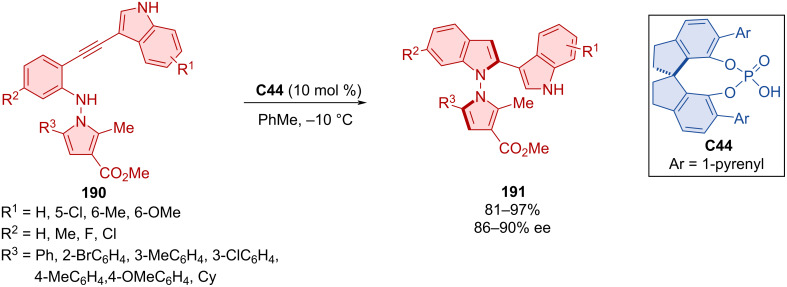
Atroposelective cyclization of 3-(arylethynyl)-1*H*-indoles.

The 5-*endo*-*dig*-cyclization reaction of *N*-pyrroloalkynylanilines **190** catalyzed by SPINOL-derived CPA **C44** was utilized in the formation of axially chiral products **191** (Scheme 56) [86]. The authors achieved good results in terms of enantioselectivities and yields with mostly methyl, methoxy, or halogen modifications in various positions. The configurational stability experiments of products **191** confirmed stable enantiopurity at 130 °C for 48 h in toluene. Investigating the biological activity for a number of compounds, good cytotoxicity was reported for five kinds of cancer cells. The mechanistic study suggests that the indole ring of the substrate is having a crucial role in the reaction mechanism because replacing it with another aromatic moiety such as phenyl or 2-thiophenyl led to no product being formed.

**Scheme 57:**
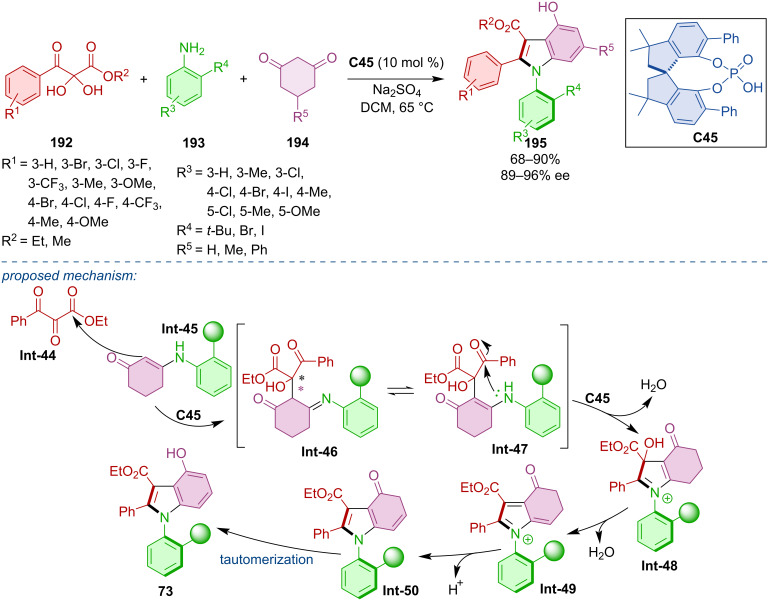
Atroposelective three-component heteroannulation.

An organocatalytic atroposelective three-component cascade heteroannulation was done by Wang et al. [87]. It was a reaction of ketoesters **192**, anilines **193**, and cyclohexadiones **194** catalyzed by SPINOL-derived CPA **C45** (Scheme 57). The authors achieved good to excellent yields and remarkable enantioselectivities and proposed a plausible reaction mechanism. The crucial step of this transformation is believed to be the asymmetric dehydrative cyclization forming biaryl intermediate **Int-48**. Subsequent dehydration, release of the chiral acid, and aromatization through tautomerization of intermediate **Int-50** generate the desired product **195**.

**Scheme 58:**
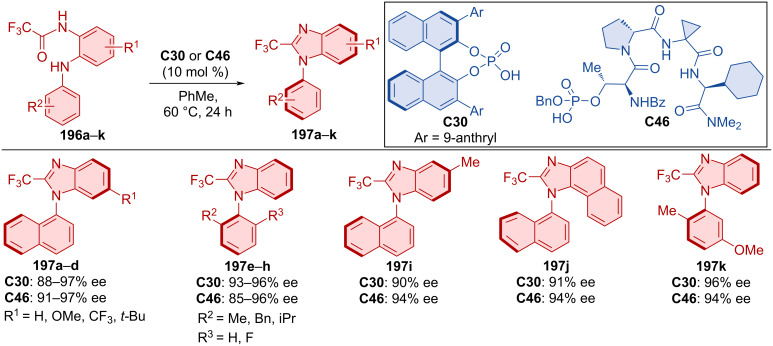
CPA-catalyzed formation of arylbenzimidazols.

Kwon et al. compared the effectivity of traditional biaryl phosphoric acid **C30** with peptide phosphoric acid **C46** in the cyclodehydration of trifluorophenylaminoacetals **196a**–**k** (Scheme 58) [88]. Both organocatalysts showed comparable effectivity in terms of enantioselectivity across. The difference between these catalysts becomes more visible through DFT calculations. In the case of biaryl phosphoric acid **C30**, stereocontrol is driven by the steric effects of the groups present. On the other hand, peptide phosphoric acid **C46** appears to work through an alternative mode of enantioinduction, where conformational adaptation presumably limits repulsive interactions.

**Scheme 59:**
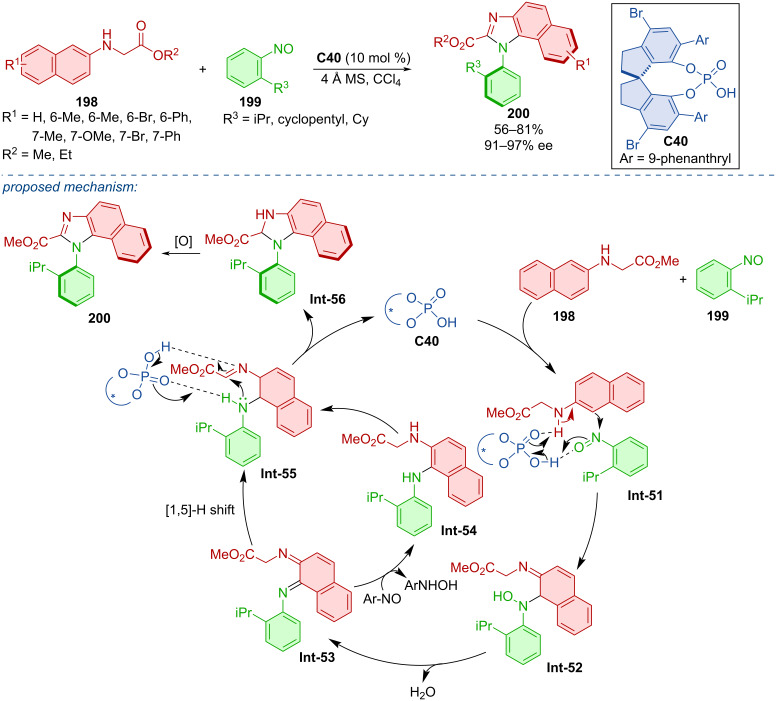
CPA-catalyzed reaction of *N*-naphthylglycine esters with nitrosobenzenes.

CPA **C40** was utilized in the construction of the imidazole ring of axially chiral products **200** (Scheme 59) [89]. *N*-Naphthylglycine esters **198** reacted with nitrosobenzenes **199** and the authors reported moderate to good yields with remarkable enantioselectivities. Configurational stability of a representative product **200** was observed in toluene at 120 °C for 24 h with no deterioration of the ee. The practicality of the developed protocol was demonstrated on a gram-scale reaction, where the corresponding product **200** was obtained with an acceptable decrease of yield and enantioselectivity (65%, 94% ee). Mechanistically, a chemo- and regioselective nucleophilic addition followed by dehydration leads to diimine intermediate **Int-53**. Control experiments confirmed that this structure could undergo successive reduction and oxidation through intermediate **Int-54** to give benzylimine intermediate **Int-55**. Alternatively, a direct [1,5]-H migration of **Int-53** also leads to **Int-55**. The stereoselectivity of the product is determined in the CPA-catalyzed intramolecular enantioselective addition and oxidative aromatization affords the final products **200**.

**Scheme 60:**
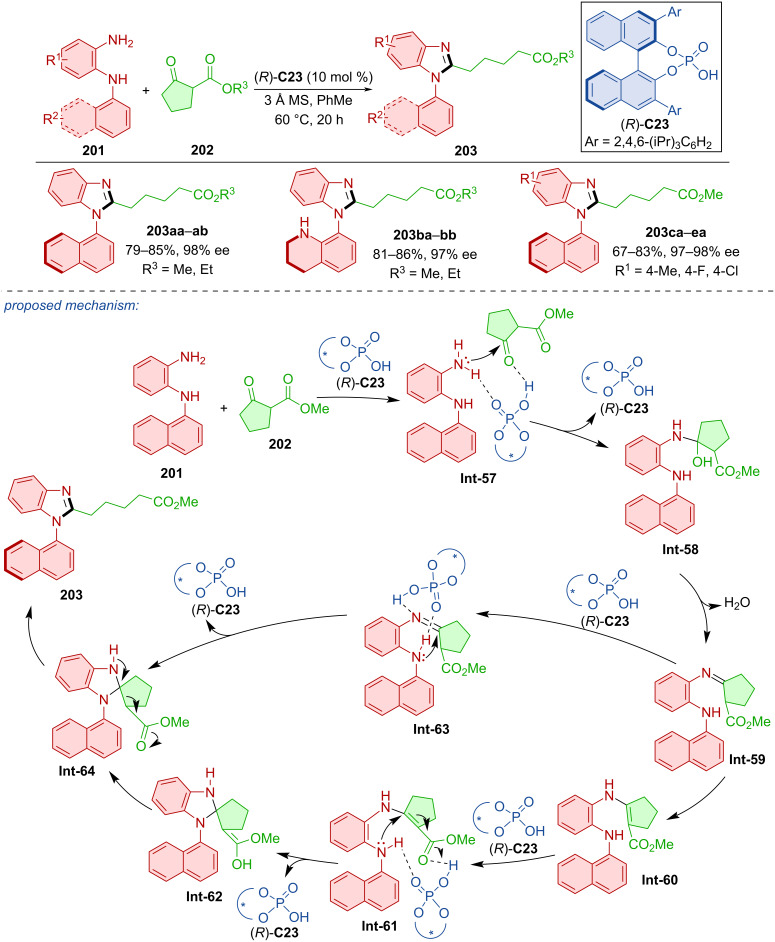
CPA-catalyzed formation of axially chiral *N*-arylbenzimidazoles.

The organocatalytic construction of the imidazole ring to provide axially chiral *N*-arylbenzimidazoles **203** was catalyzed by CPA (*R*)-**C23** (Scheme 60) [90]. It was a reaction of *N*-arylbenzenediamines **201** and β-ketoesters. Testing many different substrates, oxocyclopentane carboxylates **202** consistently provided the highest yields and enantioselectivities. The authors observed no racemization of **203** below 90 °C in toluene, isopropyl alcohol, or DCE. Based on these results, the rotational barrier was calculated to be 32.9 kcal/mol. The proposed reaction pathway starts with CPA activation of the substrates, nucleophilic addition and dehydration leading to imine intermediate **Int-59**. After dehydration to **Int-59**, two possible approaches could be utilized. The first of the two possible approaches consists of isomerization and another catalyst activation promoting an intramolecular Michael addition. The organocatalyst is freed, and cyclization product **Int-62** undergoes racemization to form **Int-64**. The identical intermediate **Int-64** is formed by catalyst activation and direct nucleophilic addition. In the final step of the reaction, ring opening by the C–C bond cleavage yields the desired product **203**.

**Scheme 61:**
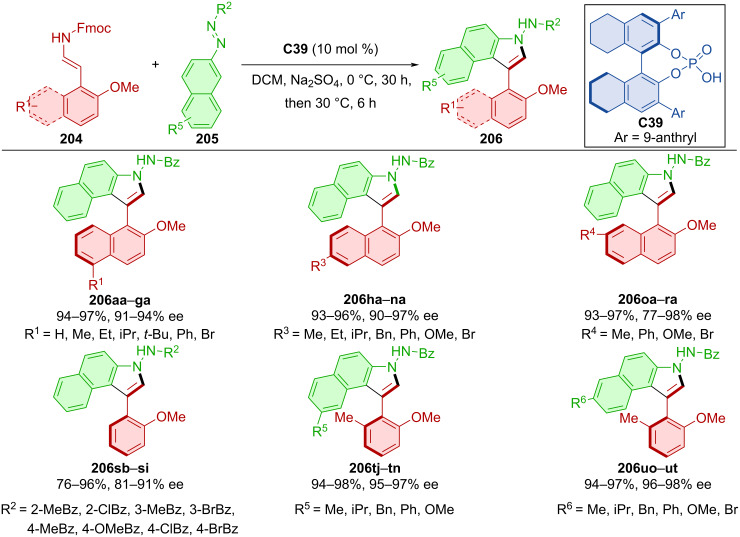
CPA-catalyzed formation of axially chiral arylbenzoindoles.

The first phosphoric acid **C39**-catalyzed asymmetric cycloaddition–elimination cascade reaction of 2-naphthol or phenol enamide derivatives **204** with azonaphthalenes **205** was done by Xu et al. in 2021 (Scheme 61) [91]. After testing a considerable number of substrates, the authors achieved excellent yields and enantioselectivities. The synthetic utility of this approach stems from being able to skip additional reaction steps and thus omit reagents used in those steps, leading to increased efficiency and, by extension, atom economy. Through the thermal racemization experiment, the rotational barrier of the product **206** was calculated to be 31.1 kcal/mol at 100 °C, corresponding to a half-life of 107 years at 25 °C.

**Scheme 62:**
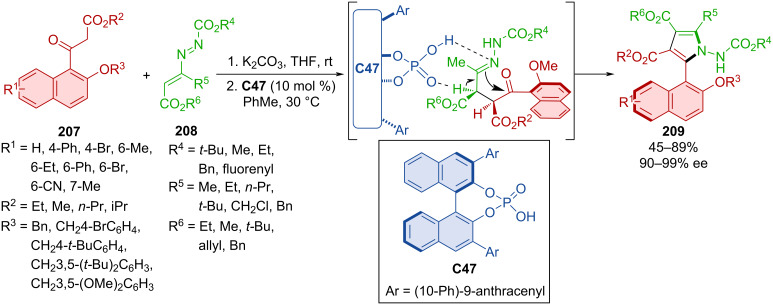
CPA-catalyzed formation of pyrrolylnaphthalenes.

Han et al. developed a protocol to construct pyrrole rings from 1-naphthyl ketoesters **207** and azoalkenes **208** catalyzed by CPA **C47** forming the axially chiral products **209** (Scheme 62) [92]. The majority of the products were obtained in very good yields and nearly all possessed excellent enantiomeric purities. Racemization experiments were carried out in *m*-xylene and toluene. The rotational barrier of several of products were determined between 30.8 and 32.8 kcal/mol which classifies them as stable class-3 atropoisomers. DFT calculations suggest that the stereoinduction is guided by the hydrogen bonds between catalyst and condensed substrates.

**Scheme 63:**
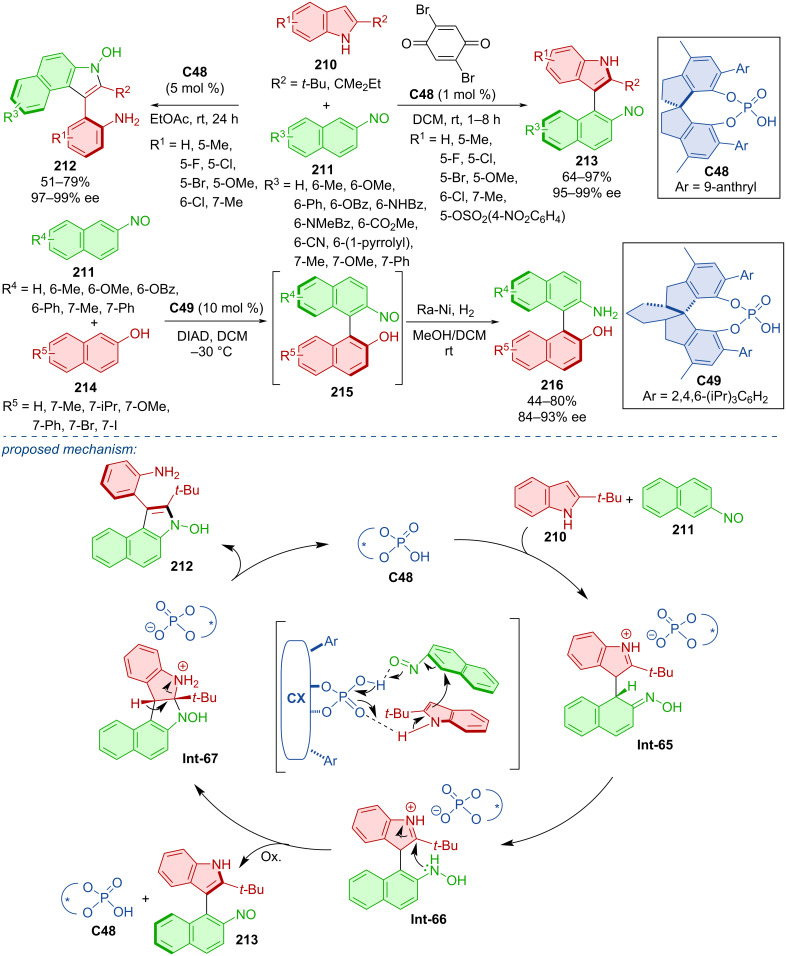
CPA-catalyzed addition of naphthols and indoles to nitronaphthalenes.

A DFT-guided study of the CPA **C48**-catalyzed reaction of indoles **210** with 2-nitrosonaphthalenes **211** was conducted in 2020 (Scheme 63) [93]. In combination with catalyst hydrogen bonding, the nitroso group was identified as a suitable partner for the nucleophilic substitution by possessing a sufficiently low LUMO energy value. Based on the reaction conditions, two different products were formed. Arylbenzoindoles **212** by standard conditions with 5 mol % catalyst loading and naphthylindoles **213** by utilization of an oxidant with only 1 mol % catalyst loading. Under these optimized conditions, decent yields and remarkable enantioselectivities were achieved. 2-Nitrosonaphthalenes **211** could also react with 2-naphthols **214** in the presence of CPA **C49** to form axially chiral binaphthyls **215** and finally after hydrogenation atroposelective NOBINs **216**. This reaction yielded products in mostly moderate amounts with good levels of enantiomeric purity. Transition-state calculations gave insight into possible reaction pathways. CPA **C48**-activated substrates react and rearomatization of the benzene ring leads to the intermediate **Int-66**. In the presence of an oxidant, axially chiral product **213** is formed. Otherwise, nitrogen-initiated intramolecular cyclization takes place, subsequent β-H elimination, and C–N-bond cleavage lead to the axially chiral indolylaniline **212**.

**Scheme 64:**
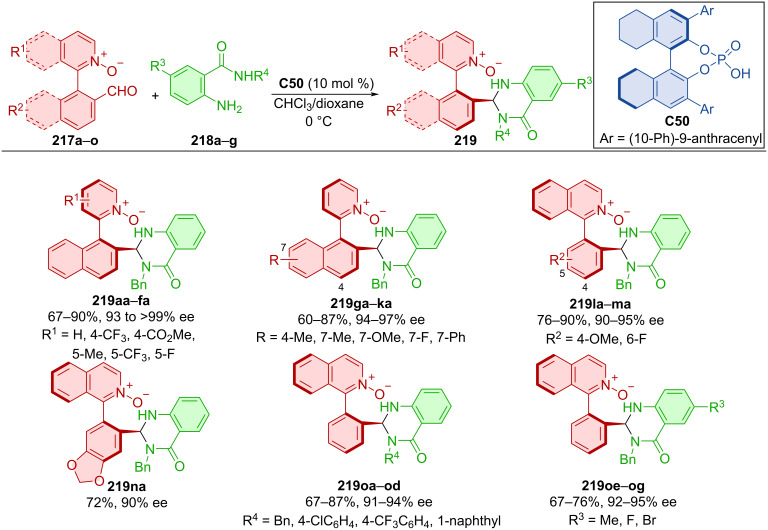
Atroposelective reaction of heterobiaryl aldehydes and aminobenzamides.

Heterobiaryl aldehydes **217a**–**o** and aminobenzamides **218a**–**g** reacted in the presence of CPA **C50** leading to axially chiral products **219** (Scheme 64) [94]. Investigating various combinations of naphthyl and phenyl substituents provided satisfactory to good yields in general with high degrees of enantiomeric purity. Scaling up the reaction to a one-mmol scale with reduced catalyst loading of 5 mol % resulted in high yield and slightly decreased enantiomeric purity for the representative product **219oa** (84%, 92% ee).

**Scheme 65:**
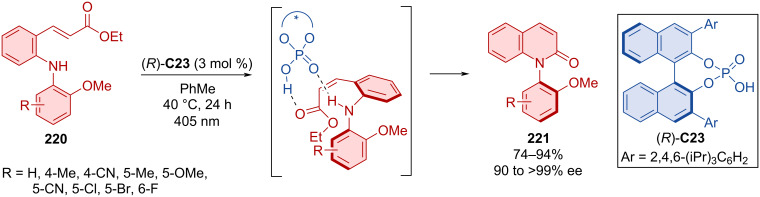
Atroposelective cyclization forming *N*-arylquinolones.

The unique combination of photochemistry and Brønsted acid-catalysis by CPA (*R*)-**C23** was utilized in the cyclization reaction of cinnamates **220** forming *N*-arylquinolones **221** (Scheme 65) [95]. Optimized reaction conditions led to the formation of products **221** in remarkable yields with astounding enantioselectivities. Both, light and organocatalyst proved crucial for the reaction. The photochemical aspect isomerizes the double bond to (*Z*)-configuration, and CPA stabilizes the structure whilst mediating the cyclization. The wavelength of 405 nm was chosen so that only the starting material, not the organocatalyst nor the product being formed, effectively absorb the light.

**Scheme 66:**
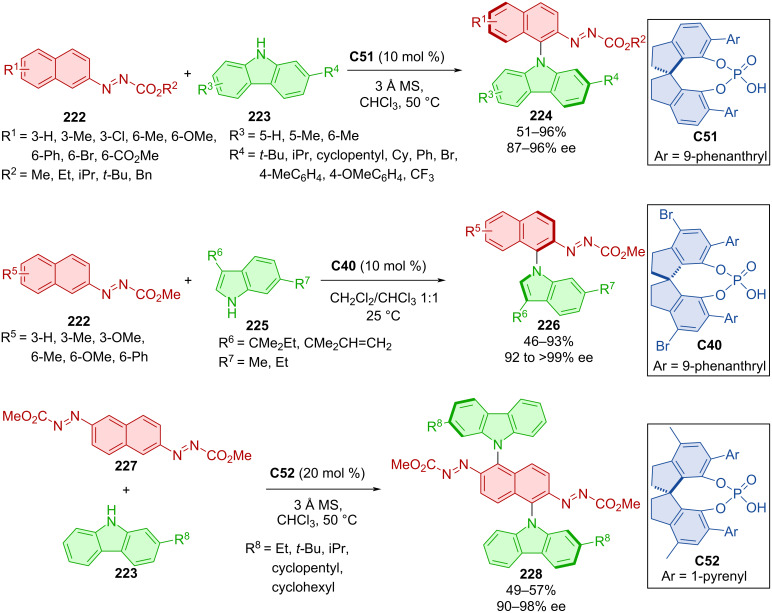
Atroposelective formation of 9*H*-carbazol-9-ylnaphthalenes and 1*H*-indol-1-ylnaphthalene.

An atroposelective C–H amination was done with the help of SPINOL-derived chiral phosphoric acids **C51**, **C40**, and **C42** (Scheme 66) [96]. It was a reaction of naphthalenyldiazene carboxylates **222** with derivatives of carbazole **223** or indole **225**. The reactions provided excellent enantioselectivities and good yields and the best results were achieved with methyl or chlorine substituents in position 3 of the naphthyl ring. Dicarbazoles are generally useful as OLED materials [97]. For this reason, the authors decided to apply the optimized reaction conditions to prepare such compounds. Dicarbazoles **228** were prepared in moderate yields with high enantiomeric purity.

**Scheme 67:**
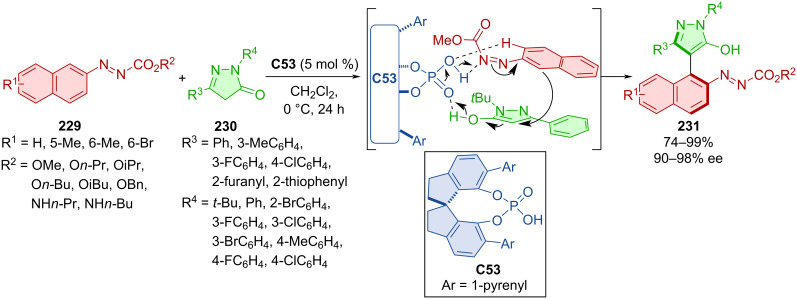
CPA-catalyzed formation of pyrazolylnaphthalenes.

Azonaphthalenes **229** readily reacted with pyrazoles **230** in the presence of CPA **C53** giving access to axially chiral products **231** (Scheme 67) [98]. In the majority of the experiments, great to near-perfect yields and enantioselectivities were reported. The racemization experiment using product **231** in iPrOH at 80 °C resulted in a rotational barrier of 27.3 kcal/mol, which is in good agreement with the calculated value (26.7 kcal/mol). DFT calculations suggest the crucial role of CPA catalyst in the activation of the reaction through corresponding hydrogen bonds.

Gao et al. developed a method of dynamic kinetic resolution providing axially chiral products in good yields, enantio- and diastereoselectivities, but with low rotational barriers (26 kcal/mol) [99].

**Scheme 68:**
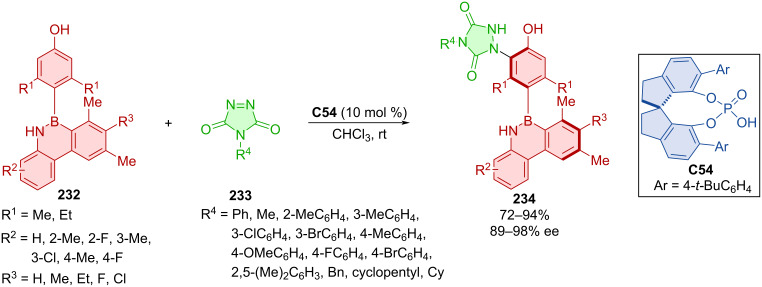
Atroposelective addition of diazodicarboxamides to azaborinephenols.

Yang et al. developed a SPINOL-derived CPA **C54**-catalyzed electrophilic substitution of diazodicarboxamides **233** and azaborinephenols **232** leading to axially chiral products **234** (Scheme 68) [100]. Excellent enantioselectivities and very good yields were reported in almost all cases. More modest yields were reported with different R^3^ substituents. The preparative utility of this protocol was demonstrated in the gram-scale reaction providing product **234** in almost identical yield and enantiomeric purity with only 5 mol % catalyst loading (85%, 94% ee). A control experiment without catalyst provided the product (75%), but at a significantly slower rate than the catalyzed one. Methylation of the oxygen in azaborinephenol **232** led to no product being formed. On the other hand, methylation of nitrogen in azaborinephenol **232** still provided the product in decent yield and enantioselectivity (79%, 89% ee) albeit with longer reaction time. A representative axially chiral azaborine exhibited good configurational stability even at 120 °C for 24 h without obvious racemization.

**Scheme 69:**
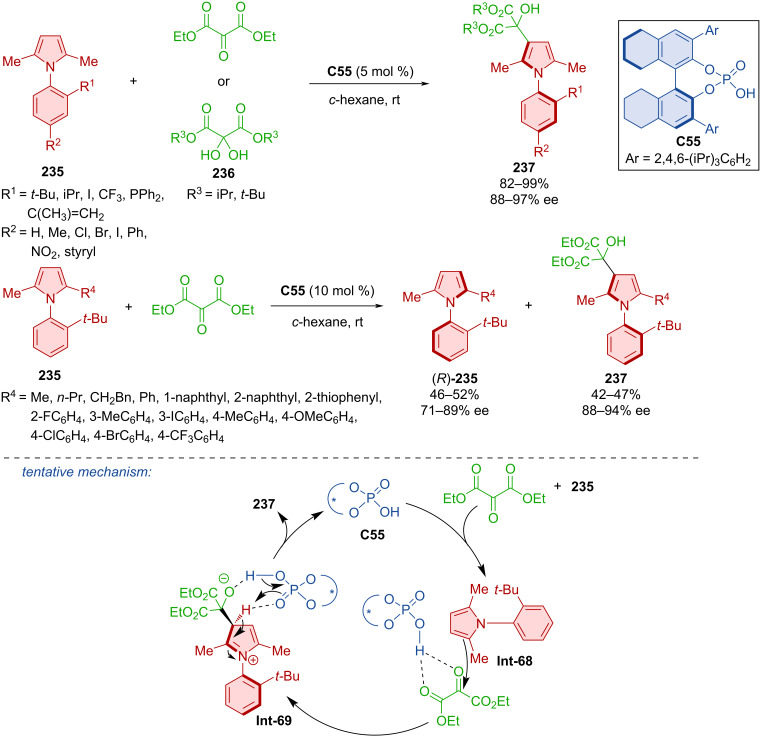
Catalytic formation of axially chiral arylpyrroles.

Zhang et al. utilized desymmetrization and kinetic resolution with CPA **C55** in two complementary approaches to prepare atropoisomeric arylpyrrole derivatives (*R*)-**235** and **237** [101]. In the first case, arylpyrroles **235** reacted with either diethyl 2-oxomalonate or dihydroxymalonate ester derivatives **236** (Scheme 69). A kinetic resolution was done with arylpyrroles **235** and diethyl 2-oxomalonate. The possibility of the kinetic resolution was discovered when authors used asymmetrical arylpyrroles **235** instead of symmetrical ones. Both approaches proved effective with high yields and excellent enantioselectivities. Literature research and experimental results gave insight into the potential mechanism of the reaction [102–103]. Hydrogen bonds between the ketomalonate and organocatalyst **Int-68** were shown as the pivotal interaction that formed the chiral pocket for the induction of chirality. Nucleophilic addition followed by rearomatization of the pyrrole ring and protonation of the oxygen forms the axially chiral arylpyrrole **237**.

**Scheme 70:**
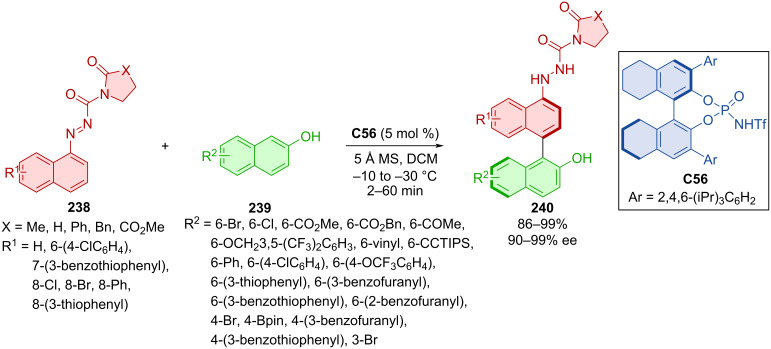
Atroposelective coupling of 1-azonaphthalenes with 2-naphthols.

The organocatalytic cross-coupling reaction of 1-azonaphthalenes **238** with 2-naphthols **239** catalyzed by chiral *N*-triflylphosphoramide **C56** was done in 2023 (Scheme 70) [104]. A remarkable number of axially chiral products **240** were prepared with excellent enantiomeric purities and high yields. Undiminished yields and enantioselectivities (99%, 94% ee) were observed in a gram-scale experiment with only 2 mol % of the organocatalyst **C56**.

**Scheme 71:**
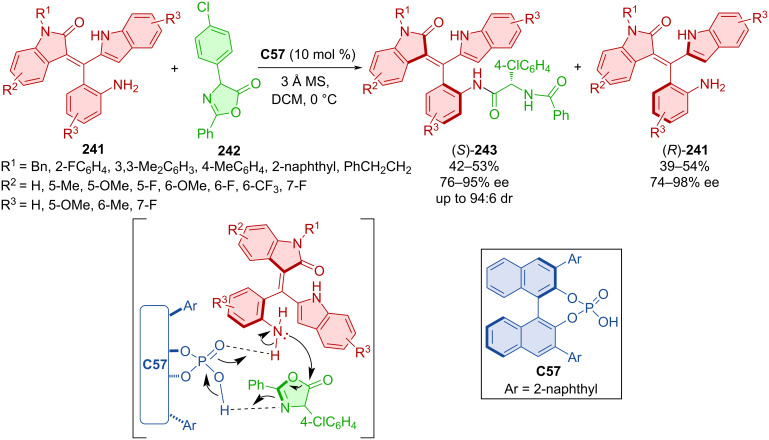
CPA-catalyzed formation of axially chiral oxindole-based styrenes.

The CPA **C57**-catalyzed kinetic resolution was utilized in the formation of axially chiral oxindole-based styrenes (*S*)-**243** and (*R*)-**241** (Scheme 71) [105]. On top of good enantioselectivities and decent yields, very good diastereomeric ratios (up to 94:6) were achieved. The suggested stereoinduction is shown below, with CPA forming hydrogen bonds and activating both substrates. The products incorporated into a thiourea organocatalyst were utilized in (4 + 2) and (3 + 2) annulation reactions providing moderate to high yields, low enantioselectivities, but excellent diastereomeric ratios (57–91%, 32–41% ee, >95:5 dr).

Shao, Cheng, and co-workers developed an atroposelective synthesis of axially chiral styrene-type allylamines [106]. A chiral phosphoric acid organocatalyst catalyzed the key transformation, the reductive amination of 1-enal-substituted 2-naphthols.

**Scheme 72:**
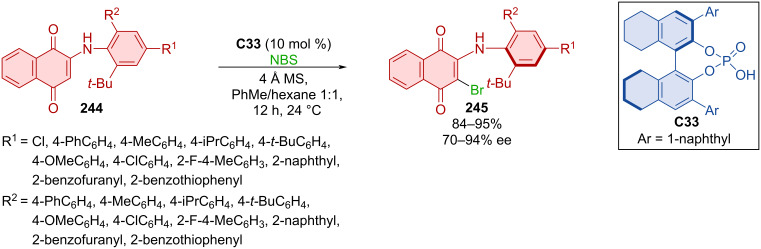
Atroposelective electrophilic bromination of aminonaphthoquinones.

Vaidya et al. developed an atroposelective organocatalytic electrophilic halogenation of aminonaphthoquinones **244** with NBS catalyzed by biaryl catalyst **C33** (Scheme 72) [107]. This seemingly two-axes system is simplified into a single-axis system by a strong intramolecular N–H–O hydrogen bond. Enantiopurities and yields of products **245** were very high. Racemization studies showed that product **245** is a stable class-3 atropoisomer with a barrier of rotation of 30.3 kcal/mol at 100 °C in toluene. Testing in ethanol at 80 °C and in strongly acidic conditions (EtOH/0.5 M HCl 4:1) provided only a slight decrease to 29.1 kcal/mol and 28.9 kcal/mol, respectively, or remained largely unchanged at 30.6 kcal/mol with toluene under buffered aqueous conditions (pH 7.5 in 1 M Tris buffer). These findings suggest this compound would be stereochemically stable in biological environments, showing potential usefulness in medicinal chemistry.

**Scheme 73:**
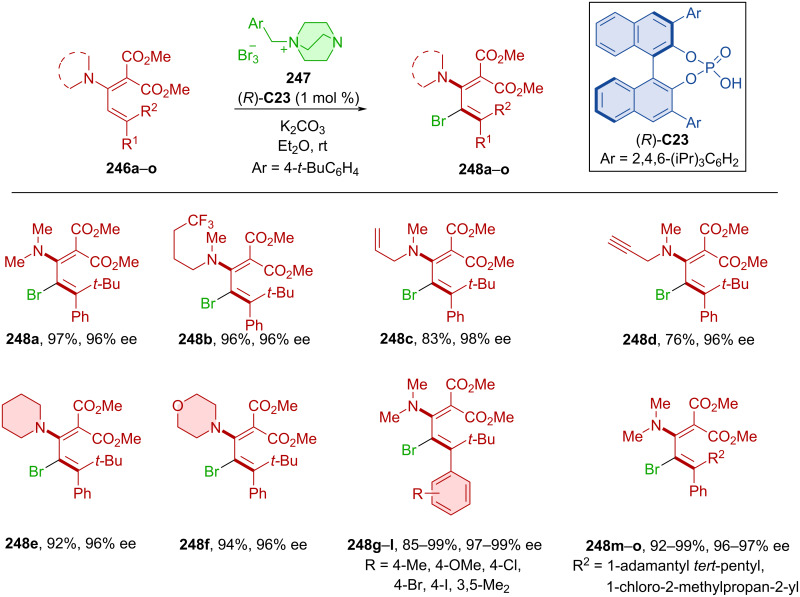
Atroposelective bromination of dienes.

A peculiar class of atropoisomers was prepared in 2024 by bromination catalyzed by CPA (*R*)-**C23** (Scheme 73) [108]. The DABCO-derived cationic bromination agent **247** was used with dienes **246a**–**o** to form axially chiral dienes **248a**–**o**. Considerable substrate scope demonstrated via a wide range of products **248a**–**o** in high yields and remarkable enantiomeric purities. Bromine could subsequently be reacted further, expanding on the synthetic utility of the products. The authors ascertained that the Grignard exchange reaction with PhMgCl could reconvert products **248a**–**o** into reactive nucleophilic intermediates. The rotational barrier of the product **248a** was determined 36.4 kcal/mol by DFT calculations and 35.5 kcal/mol experimentally.

**Scheme 74:**
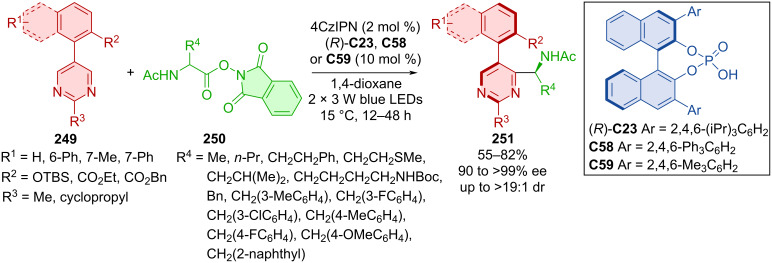
CPA-catalyzed formation of axially chiral 5-arylpyrimidines.

A dual photoredox and CPA-catalyzed process was developed for the atroposelective construction of axially chiral 5-arylpyrimidines **251** [109]. The strategy relied on a Minisci-type reaction of 5-arylpyrimidines **249** and α-amino acid-derived redox-active esters **250**. This transformation was enabled by 4CzIPN as an organic photoredox catalyst in conjunction with a chiral phosphoric acid catalysts (*R*)-**C23**, **C58**, or **C59** (Scheme 74).

**Scheme 75:**
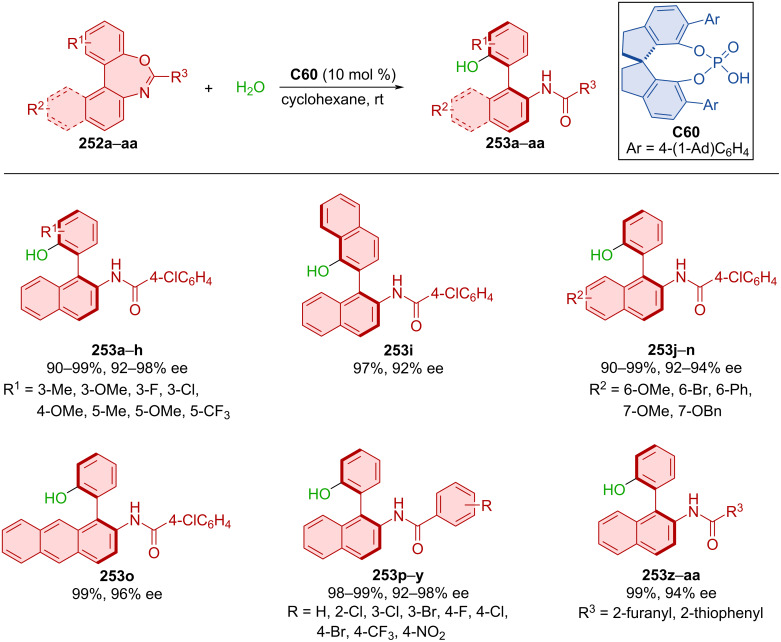
Atroposelective hydrolysis of biaryloxazepines.

CPA **C60** catalyzed the asymmetric hydrolysis of biaryloxazepines **252a**–**aa** leading to the formation of axially chiral biarylamides **253a**–**aa** (Scheme 75) [110]. This method proved to be a reliable strategy providing axially chiral products **253a**–**aa** in excellent yields and enantiomeric purities. Starting from racemic **253** by subsequent Mitsunobu reaction and CPA-catalyzed asymmetric hydrolysis on a 2.0 mmol scale reaction, a comparably high yield and enantioselectivity was achieved (95%, 96% ee).

**Scheme 76:**
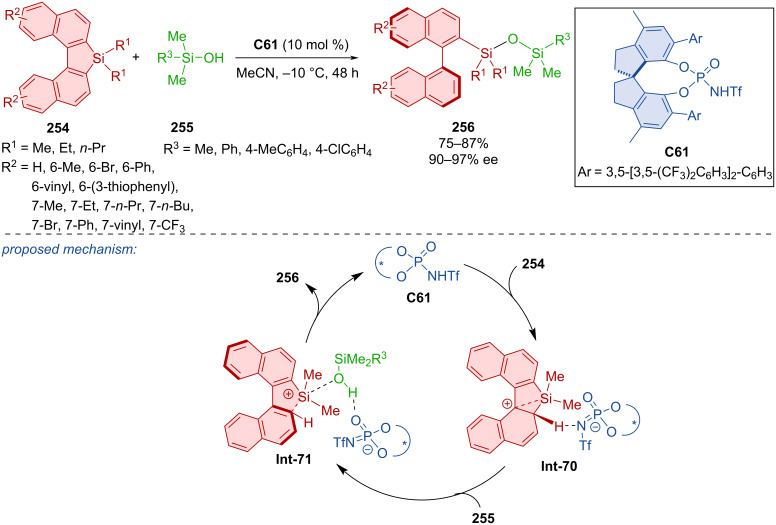
Atroposelective opening of dinaphthosiloles.

The unique preparation of axially chiral biarylsiloxanes **256** from dinaphthosiloles **254** and silylalcohols **255** catalyzed by chiral *N*-triflylphosphoramide **C61** was first done in 2023 (Scheme 76) [111]. Exceptional enantioselectivities and very good yields were achieved with many different dinaphthosiloles containing groups in positions 6 and 7 and a number of silylalcohols as well as in a gram-scale synthesis (90%, 91% ee). The synthetic utility of the reaction stems from efficient atom economy, scalability, operational simplicity, mechanistic novelty, and preparation of axially chiral ligands. The authors presented a proposed reaction mechanism substantiated by DFT calculations. The chiral organocatalyst **C61** forms intermediate **Int-70** stabilized by β-sillicon effect. Subsequent addition, deprotonation, and ring opening leads to the formation of axially chiral biaryl siloxane **256**.

**Scheme 77:**
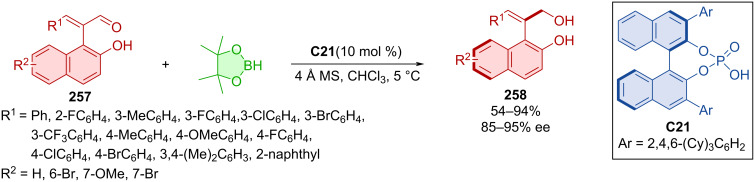
Atroposelective reduction of naphthylenals.

Jiang et al. utilized phenylacrylaldehydes **257** and pinacolborane in combination with CPA **C21**, promoting asymmetric hydrogenation to obtain axially chiral aryl allylalcohols **258** (Scheme 77) [112]. The reaction afforded a wide range of products **258** in synthetically relevant yields with very high enantioselectivities. Heating in isopropanol at 60 °C led to a decrease in ee values and the experimentally calculated racemization barrier was set to 27.9 kcal/mol.

### Hydrogen-bond-donating catalysts

**Scheme 78:**
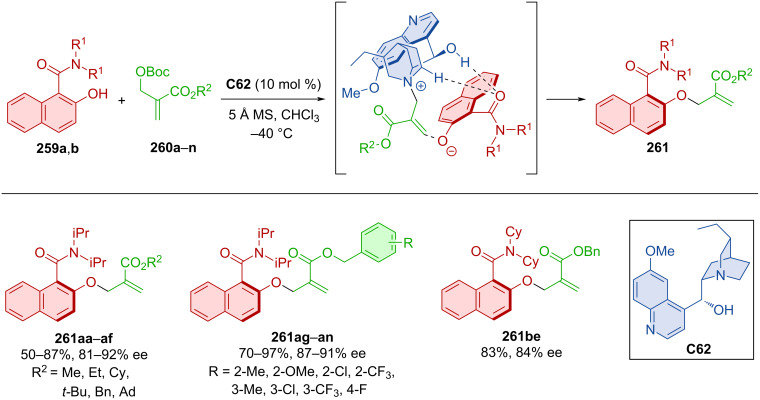
Atroposelective allylic substitution with 2-naphthols.

Hydroquinine **C62** was utilized in the dynamic kinetic resolution of racemic naphthylamides **259a**,**b** by atroposelective alkylation with carbonates **260a**–**n** forming axially chiral products **261** (Scheme 78) [113]. Good to high yields and excellent enantioselectivities were achieved with a slight decrease of enantioselectivity (66 and 48% ee) on substrates with a methyl or methoxy group in position 4 of 2-naphthol. Comparable results were achieved in the gram-scale preparation of product **261ae**, achieving >99% ee after recrystallization. The organocatalyst interacts with the substrates through hydrogen bonds with the oxygen of the naphthylamide and covalent interaction with carbonate.

**Scheme 79:**
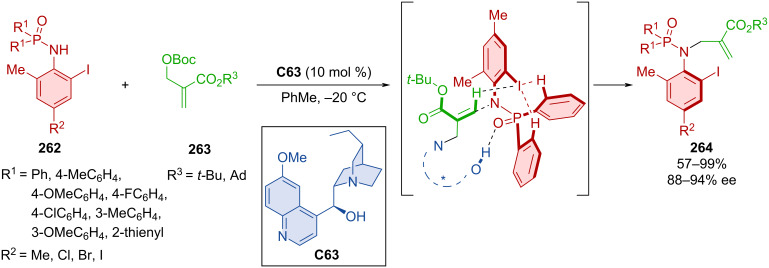
Atroposelective allylic alkylation with phosphinamides.

Yang et al. developed an asymmetric atroposelective *N*-alkylation of phosphinamides **262** with carbonates **263** catalyzed with hydroquinidine **C63** forming axially chiral *N*-alkylated phosphinamides **264** (Scheme 79) [114]. Moderate to high yields and very good enantioselectivities were reported utilizing the optimized reaction conditions. DFT calculations suggest that stereocontrol is derived from a weak hydrogen bond between iodine in the *ortho* position and other hydrogens on sp^2^ carbons throughout both substrates. The opposite enantiomer would require the methyl group to vacate this chiral pocket, which would cause steric repulsion. This unfavorable transition state was calculated to have a 2.0 kcal/mol higher energy than the favorable one. Hypervalent iodine(III) present in the products could be used to catalyze the asymmetric oxidative dearomatization of phenols.

**Scheme 80:**
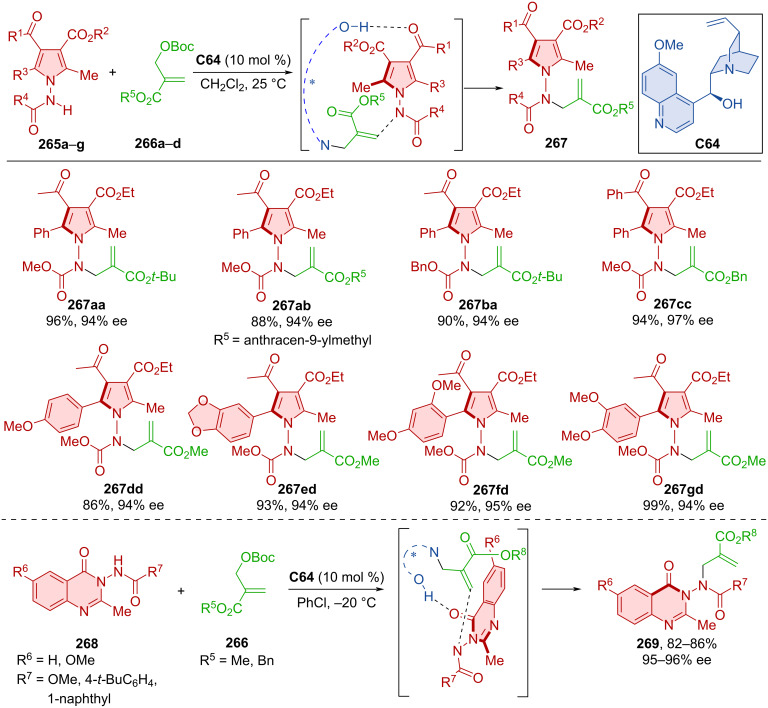
Atroposelective allylic substitution with aminopyrroles.

Atroposelective *N*-alkylations catalyzed by quinidine **C64** were also done by Mei et al. in 2021 (Scheme 80) [115]. In one reaction, carbonates **266a**–**d** were used as alkylation reagents, along with aminopyrroles **265a**–**g**. The second was the reaction of aminoquinazolinones **268** with similar carbonates **266**. Axially chiral products **267** and **269**, respectively, were prepared with very high levels of enantiopurity and high yields. Racemization experiments carried out on product **269** led to decreased ee values over time. Through these experiments and DFT calculations, rotational barriers were calculated between 29.6 and 32.3 kcal/mol.

**Scheme 81:**
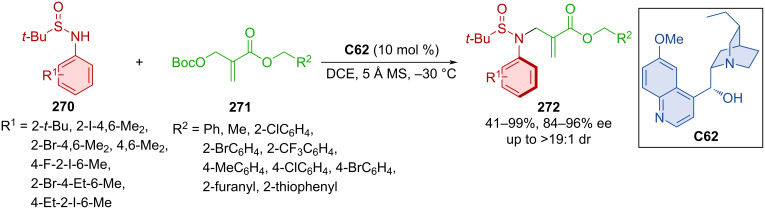
Atroposelective allylic substitution with aromatic sulfinamides.

Carbonates **271** proved suitable reaction partners for arylsulfinamides **270** in the presence of hydroquinine **C62** affording alkylated axially chiral sulfinamides **272** (Scheme 81) [116]. A wide range of moderate to high yields were achieved with consistently high enantioselectivities and mostly high diastereoselectivities. The investigation of the rotational barrier provided a good result (31.1 kcal/mol) for the chosen product **272**, indicating a half-life of up to 11.1 hours at 105 °C.

Huang et al. demonstrated one case of asymmetrically prepared selenovinylsulfones in the presence of quinine-derived squaramide in moderate yield and good enantioselectivity (43%, 84% ee) [117].

**Scheme 82:**
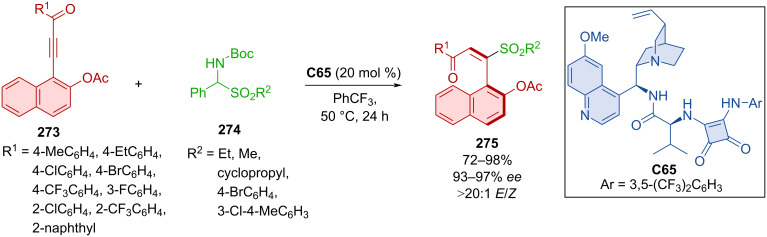
Atroposelective sulfonylation of naphthylynones.

Aminosulfones **274** acted as nucleophiles in the reaction with arylynones **273** mediated by cinchona alkaloid squaramide **C65** resulting in axially chiral products **275** (Scheme 82) [118]. In all cases, the *E*-isomer was predominant and good to excellent yields with very high enantioselectivities were reported. The model product **275** was successfully tested in reactions with a Grignard reagent and sodium tetrahydroborane resulting in the expected products.

**Scheme 83:**
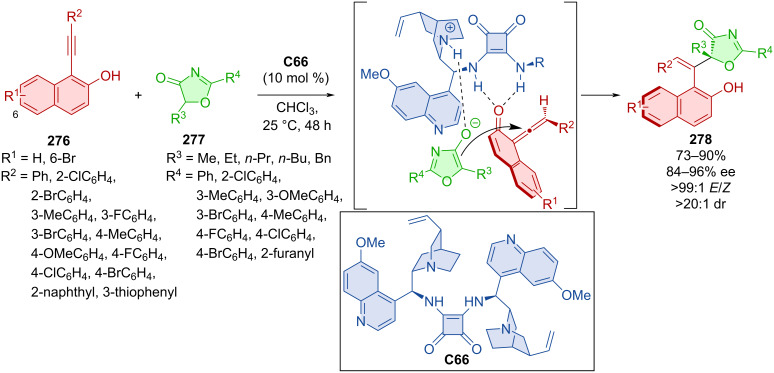
Squaramide-catalyzed reaction of alkynyl-2-naphthols with 5*H*-oxazolones.

Alkynyl-2-naphthols **276** with 5*H*-oxazolones **277** in the presence of chiral squaramide bearing quinine units **C66** were being utilized in the formation of axially chiral products **278** (Scheme 83) [119]. These unique structures containing a double bond, central and axial asymmetry were prepared in amazing yields, great enantiomeric purities, and high diastereomeric ratios as one *E*/*Z* isomer. A possible mode of activation is shown below with amino squaramide hydrogens participating in hydrogen bonds with in situ-formed VQM intermediate from alkynyl-2-naphthol. The activated oxazolone in enol form on the other hand is stabilized by the quinine nitrogen. The favorability of this approach is based upon the phenyl group of the alkynyl-2-naphthol being oriented in a way that provides decreased sterical hinderance.

**Scheme 84:**
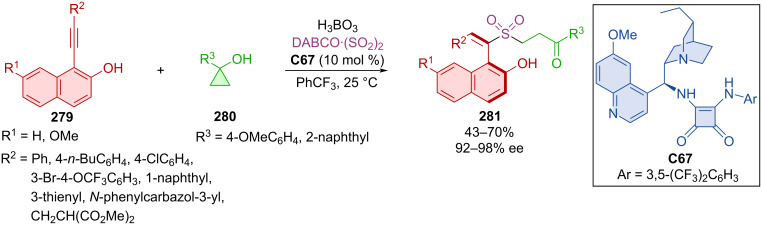
Formation of axially chiral styrenes via sulfonylative opening of cyclopropanols.

Zhang et al. developed a methodology involving cyclopropane-ring opening of **280** and alkynyl-2-naphthols **279** with the help of hydroquinine-derived squaramide **C67** leading to axially chiral products **281** (Scheme 84) [120]. Optimized reaction conditions afforded products **281** in moderate to decent yields with consistently high enantiomeric purities.

**Scheme 85:**
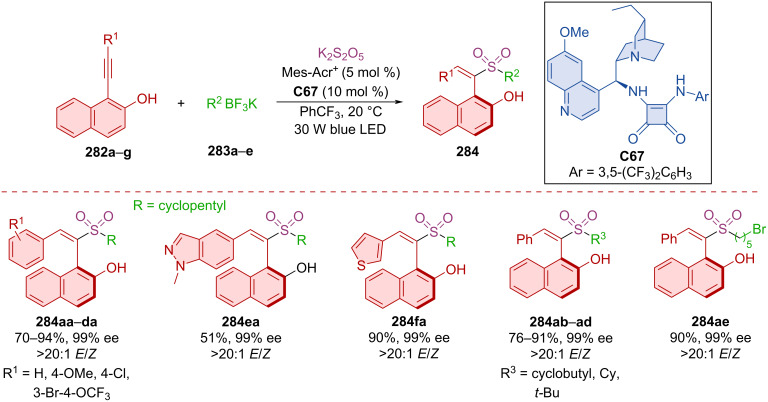
Atroposelective organo-photocatalyzed sulfonylation of alkynyl-2-naphthols.

Zhang et al. leveraged photochemical conditions, mesityl acridinium as photocatalyst and chiral squaramide **C67** to prepare axially chiral products **284** starting from alkynyl-2-naphthols **282a**–**g**, alkyl fluoroborates **283a**–**e** and a source of sulfur dioxide (Scheme 85) [121]. A wide variety of products **284** were separated as single enantiomers with great diastereoselectivity in moderate to very good yields. Demonstrating the practicality of the method, the gram-scale synthesis of a corresponding product **284** resulted in almost no deterioration in chemical yield and enantioselectivity (84%, 99% ee). A plausible radical process has been proposed based on observations and computational results.

Wada et al. contributed with their quinine-derivative-catalyzed enantioselective bromination of axially chiral cyanoarenes, achieving mild enantioselectivities in general [122]. These precedents gave solid stepping stone for further development in this field.

**Scheme 86:**
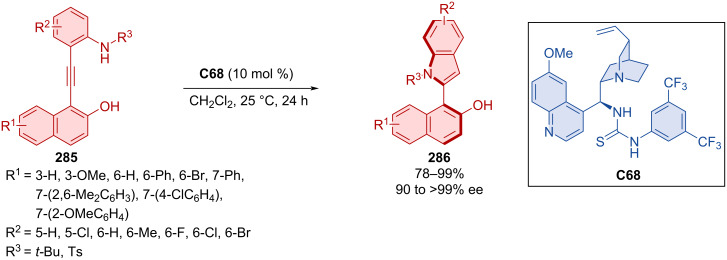
Thiourea-catalyzed atroposelective cyclization of alkynylnaphthols.

Asymmetric annulation of *o-*alkynylanilines **285** catalyzed by quinidine-derived thiourea organocatalyst **C68** led to the formation of axially chiral aryl-C2-indoles **286** (Scheme 86) [123]. High to excellent yields and enantioselectivities were achieved through optimized reaction conditions with many substrates. The transformation is presumed to occur through a VQM intermediate. After the reaction, organocatalyst **C68** could be regenerated and used again without significant loss of catalytic activity or achieved enantioselectivity. The decagram-scale reaction has led to the representative product **286** in near quantitative yield and enantiopure form (90%, >99% ee) through recrystallization without the use of column chromatography.

A chiral thiourea organocatalyst was used in asymmetric transformations leading to products in poor to moderate yields and decent enantioselectivities (17–66%, 39–80% ee) [124].

**Scheme 87:**
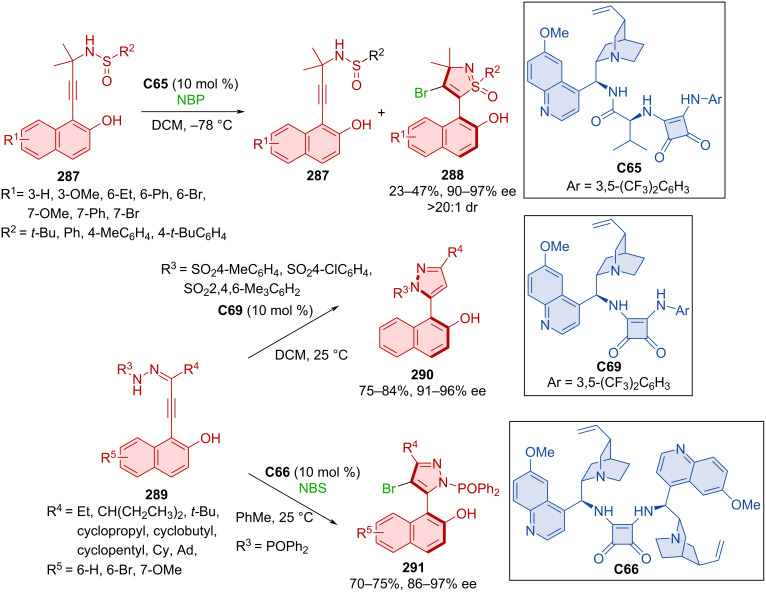
Squaramide-catalyzed formation of axially chiral naphthylisothiazoles.

Chang et al. focused their attention on the atroposelective construction of arylisothiazoles and arylpyrazoles [125]. Unique modified VQM precursor structures were designed as substrates to accomplish this task. Kinetic resolution by brominative cyclization using quinidine-derived squaramide **C65** as organocatalyst, were utilized to prepare axially chiral naphthyl-isothiazoles **288** starting from sulfinamides **287** (Scheme 87). The preparation of naphthylpyrazoles **290** and **291** was realized with quinidine-derived squaramide organocatalyst **C66** or **C69** and hydrazones **289**. Utilization of substrates bearing sulfonylbenzene groups with organocatalyst **C69** led to the formation of one enantiomer, and ones bearing a diphenylphosphine oxide group reacted with NBS and organocatalyst **C66** to provide another. Excellent enantioselectivities were achieved with all products and moderate to high yields. Some products **290** and **291** showed mild biological activity and even good antiproliferation effects during biological assays.

Cinchona alkaloid squaramide also catalyzed a double cyclization reaction through VQM leading to unique products with both helical and axial chirality [126].

**Scheme 88:**
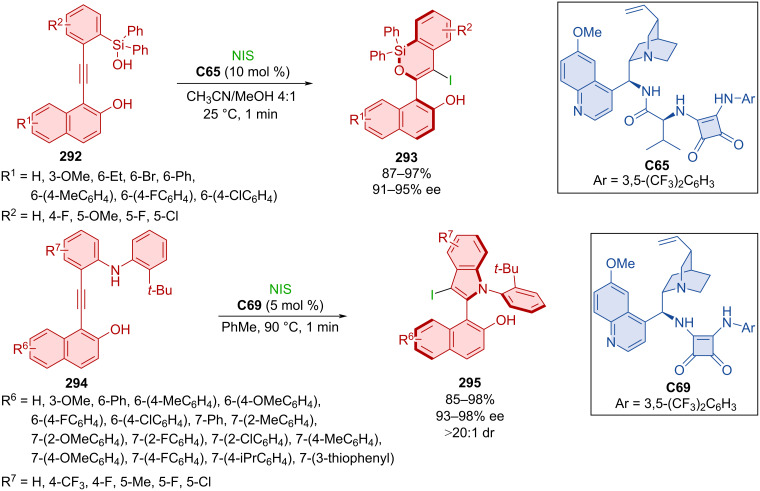
Atroposelective iodo-cyclization catalyzed by squaramide **C69**.

The fascinating work of Xu et al. demonstrated synthetic utility of alkynyl substrates in annulation reactions with the help of NIS catalyzed by quinine-based squaramides [127]. In the first case, alkynylsilanol **292** reacted to form axially chiral product **293** where organocatalyst **C65** served for chirality transfer (Scheme 88). Second was the reaction catalyzed by organocatalyst **C69** where alkynylanilines **294** formed products **295** with two stereogenic axes. High yields and high to near-perfect enantioselectivities were reported. Catalyst **C69** could be successfully utilized for up to six continuous feedings of substrate **294** and NIS to provide product **295** with good degree of enantiomeric purity. Both products **293** and **295** were also prepared in a gram-scale experiment with excellent yields and enantiomeric purities (>90%, >91% ee).

A quinine-derived thiourea organocatalyst was efficient in realizing an intramolecular reaction using VQM intermediates [128]. This atroposelective cyclization allows access to axially chiral nonsymmetric biaryltriols with up to 98% yield and 99% ee.

**Scheme 89:**
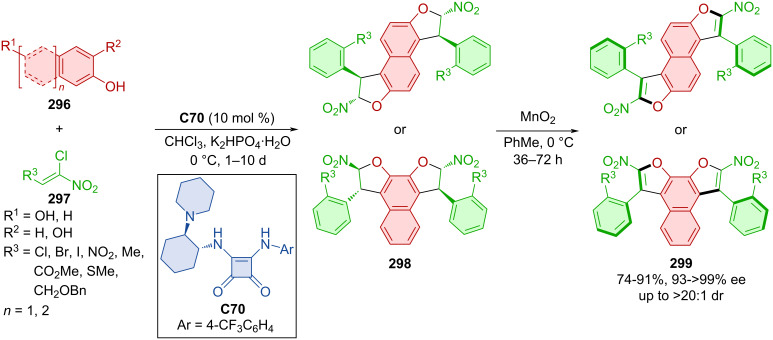
Squaramide-catalyzed formation of axially chiral oligoarenes.

Diastereomeric oligoarenes were prepared with the help of a bifunctional squaramide organocatalyst in 2020 [129]. Afterwards, MnO_2_ was successfully utilized in central-to-axial chirality conversion (Scheme 89).

**Scheme 90:**
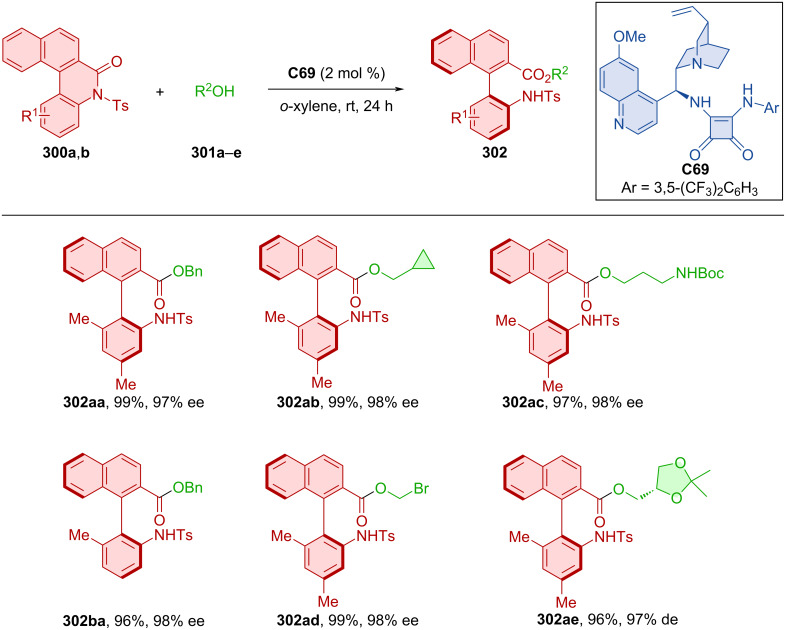
Atroposelective ring-opening of cyclic *N*-sulfonylamides.

Activation of the C–N bond of cyclic *N*-sulfonylamides **300a**,**b** with alcohols **301a**–**e** and quinidine-derived squaramide organocatalyst **C69** provided biaryl amino acid esters **302** with high levels of enantiomeric purities in near-perfect yields (Scheme 90) [130]. Stereocontrol through donor hydrogen bonds of amino squaramide groups with the oxygen on the *N*-sulfonylamide and acceptor hydrogen bond of nitrogen from quinidine to the alcohol hydrogen was proven by DFT calculations. Conformational stability was concluded by a racemization experiment, where product **302aa** could be stirred at 140 °C in *o*-xylene for 4 hours without any loss of enantiomeric purity. The rotational barrier, calculated by DFT, was 37.4 kcal/mol. Transformation of the ester group in **302aa** could lead to the formation of axially chiral amino alcohols, tripeptides, or bifunctional amines.

Using Cinchona alkaloids as catalysts, the atroposelective decarboxylative transamination of biarylaldehydes was also developed [131]. The transformation operated as a dynamic kinetic resolution and afforded the corresponding products in up to 92% yield and 66% ee.

**Scheme 91:**
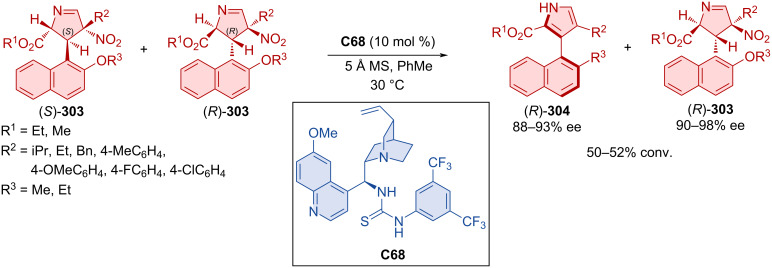
Thiourea-catalyzed kinetic resolution of naphthylpyrroles.

Zheng et al. prepared Barton–Zard intermediate (*S*,*R*)-**303** as a single diastereomer in a racemic mixture. Thiourea quinine-derived organocatalyst **C68** was then employed to promote kinetic resolution, forming axially chiral naphthylpyrroles **304** (Scheme 91) [132]. Central-to-axial chirality conversion is proposed to take place through *syn*-elimination of the nitrous acid with the help of organocatalyst **C68** and subsequent aromatization. This enantioselective aromatization applies to a broad range of substrates, leading to high enantiomeric purities and sufficient conversions. The racemization barrier of the model product (*R*)-**304** was calculated to be 34.6 kcal/mol at 140 °C, which corresponds with a half-life of one day at this temperature.

A chiral squaramide organocatalyst was also employed in a dynamic kinetic resolution of hemi-stable arylnaphthoquinones [133]. The transformation comprised atroposelective 1,4-addition of thiosugars followed by stereoretentive oxidation.

Alkenyl-substituted pyrazolone derivatives featuring an axially chiral styrene unit were obtained in high yields and with excellent diastereoselectivity and enantioselectivity [134]. The key transformation was an atroposelective addition of enolizable pyrazolones to alkynylnaphthols.

**Scheme 92:**
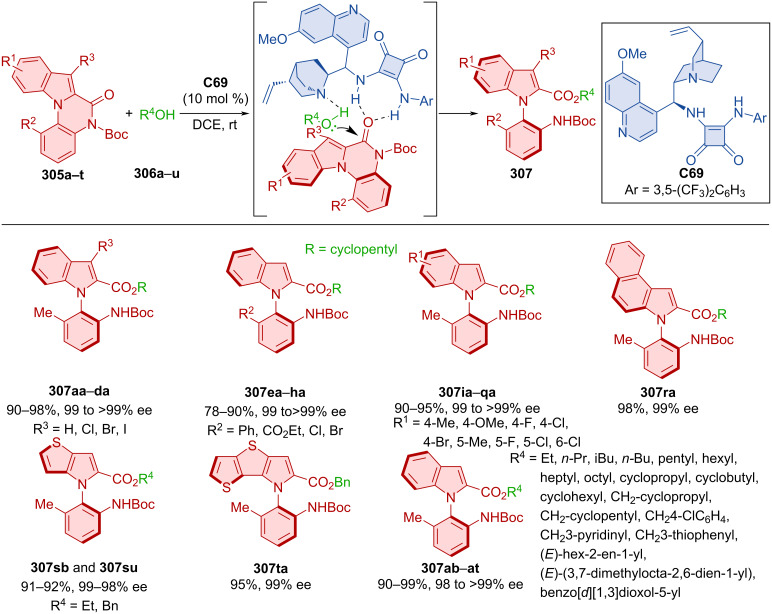
Atroposelective ring-opening of arylindole lactams.

Hong et al. utilized dynamic kinetic resolution by alcohols **306a**–**u** with *N*-Boc-*N*-arylindole lactams **305a**–**t** in the presence of bifunctional squaramide catalyst **C69** (Scheme 92) [135]. Extensive substrate scope provided an overwhelming majority of the axially chiral *N*-arylindoles **307** in near quantitative yields with next to perfect enantiomeric purity. A chosen product **307** was then tested for configurational stability in *o*-xylene at 140 °C for 12 h without any observed loss in ee. As shown below, a possible transition state consists of chiral organocatalyst **C69** providing hydrogen bonds to both substrates, dictating stereocontrol of the reaction. Demonstrating practicality of the method, gram-scale experiments provided products in comparable yields and enantioselectivities.

**Scheme 93:**
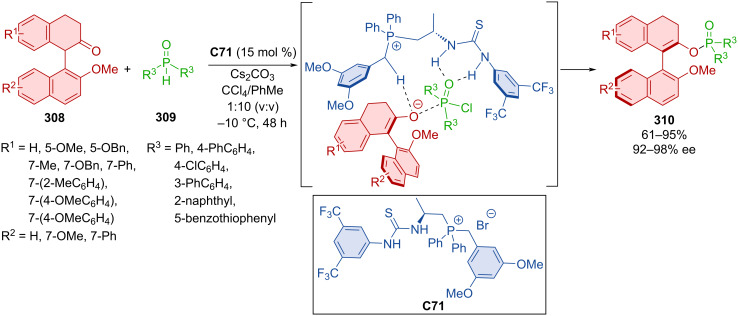
Atroposelective reaction of 1-naphthyl-2-tetralones and diarylphosphine oxides.

Fang et al. successfully carried out the Atherton–Todd reaction of 1-naphthyl-2-tetralones **308** and diarylphosphine oxides **309** catalyzed by thiourea phosphonium salt phase-transfer catalyst **C71** forming axially chiral phosphates **310** (Scheme 93) [136]. The atropoisomeric product **310** stirred in anisole at 160 °C proved to have a half-life of the enantiopurity around 24 h. The rotational barrier of this process was around 36.4 kcal/mol. Investigation of the synthetic utility of the reaction on the gram-scale led to the product **310** with comparable yield and enantiomeric purity (82%, 92% ee). Performed DFT calculations led to the proposition of a potential intermediate responsible for enantiocontrol. Thiourea amino groups provide donor hydrogen bonds to the phosphine oxide oxygen atom. Another stabilizing interaction potentially occurs between tetralone oxygen and phosphonium adjacent hydrogen on the organocatalyst.

**Scheme 94:**
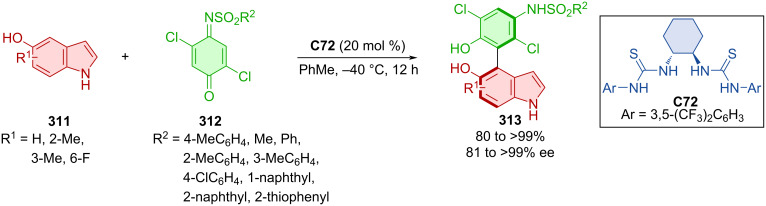
Atroposelective reaction of iminoquinones with indoles.

Iminoquinones **312** as widely applicable reaction partners in arylation reactions, where they also play a role of oxidants, were utilized in the reaction with 5-hydroxyindoles **311** (Scheme 94) [137]. The authors present the first case of axially chiral arylindoles **313** being prepared with thiourea organocatalyst **C72**, omitting usage of CPAs, which were utilized much more in the past. This approach led to near-perfect yields and enantioselectivities in best cases, with great results being achieved on the lower end as well. Chosen product **313** was tested as chiral ligand in a model reaction, providing the corresponding product in good results with a high degree of enantiomeric purity (87%, 87% ee). NMR experiments suggested the importance of hydrogen bonding in the reaction mechanism. Furthermore, in large-scale experiments (90%, >99% ee), organocatalyst **C72** was repeatedly (up to 5-times) recovered after the reaction with excellent yields (98–99%).

### Other types of organocatalysts

**Scheme 95:**
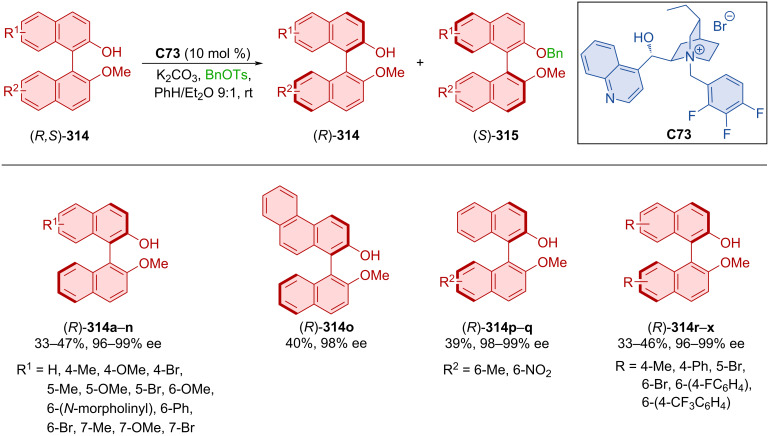
Kinetic resolution of binaphthylalcohols.

Phase-transfer catalyst **C73** was utilized in the kinetic resolution of BINOLs (*R,S*)-**314** through benzylation by benzyl tosylate affording resolved axially chiral (*R*)-**314** and benzylated axially chiral (*S*)-**315** (Scheme 95) [138]. Slightly better enantioselectivities were achieved with unprotected BINOLs (*R*)-**314** than with benzylated ones. The practicality of the process was demonstrated on a 22 mmol scale reaction with comparable results for both products (**314** – 98% ee and **315** – 80% ee). In accordance with literature, the authors proposed that the mechanism takes place through rapid and reversible deprotonation followed by slow conversion for the disfavored and fast conversion for the favored enantiomer.

**Scheme 96:**
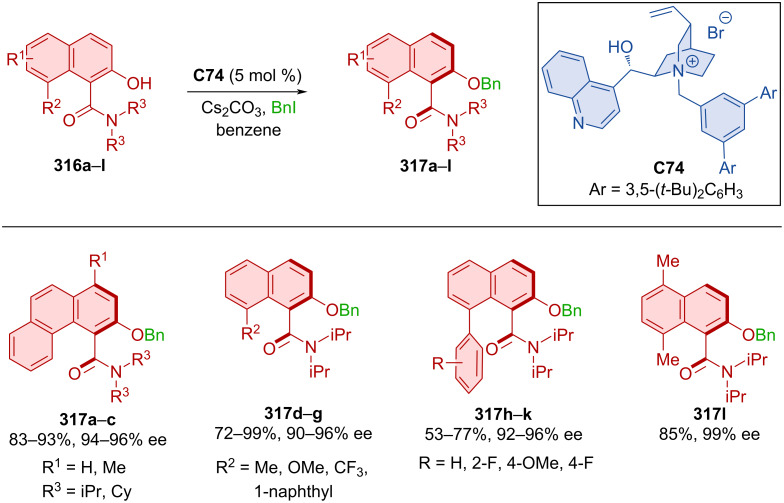
DKR of hydroxynaphthylamides.

Fugard et al. developed a highly enantioselective route to axially chiral naphthamides **317a**–**l** (Scheme 96) [139]. Through dynamic kinetic resolution by benzylation of racemic naphthamides **316a**–**l** catalyzed with phase-transfer organocatalyst **C74**, moderate to high yields with excellent enantioselectivities were achieved. The utilization of aromatic rings in position 8 of the naphthamide led to a slight decrease in yields (**317g**–**k**). The rotational barrier of starting material **316l** containing a hydroxy group was calculated to be 22.8 kcal/mol. On the other hand, the benzylated product **317l** had a rotational barrier of 31.4 kcal/mol. The authors suggest that the high rotational barrier of the starting material is due to the intramolecular hydrogen bond between the hydroxy group and amide nitrogen. This interaction was then used to promote dynamic kinetic resolution providing products with even higher rotational barrier.

**Scheme 97:**
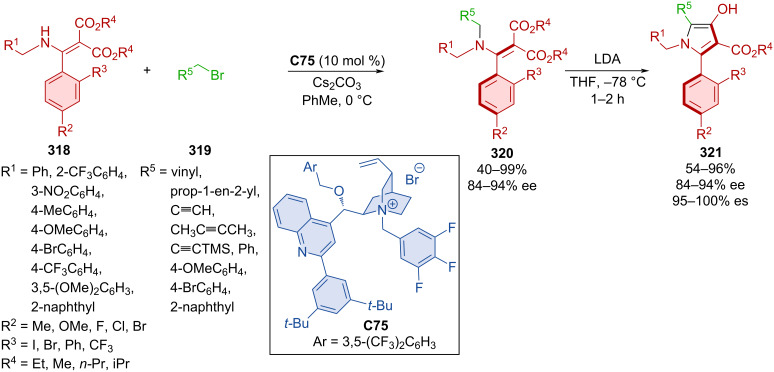
Atroposelective *N*-alkylation with phase-transfer catalyst **C75**.

Phase-transfer quinine-based organocatalyst **C75** was utilized in the transformation of arylenamines **318** with aliphatic and aromatic bromo derivatives **319** that led to axially chiral amino esters **320** (Scheme 97) [140]. The addition of LAH subsequently led to the cyclization and formation of a pyrrole ring providing axially chiral 2-arylpyrroles **321** in good to high yields and very good enantioselectivities retaining stereoinformation from the previous step. The possibility for a one-pot two-step reaction protocol was explored with satisfying yield and enantioselectivity of the corresponding product (83%, 93% ee).

A phase-transfer catalyst was also utilized in dynamic kinetic resolution by benzylation resulting in products with good yields, but moderate enantiomeric purities (53–86%, 40–80% ee) [141].

**Scheme 98:**
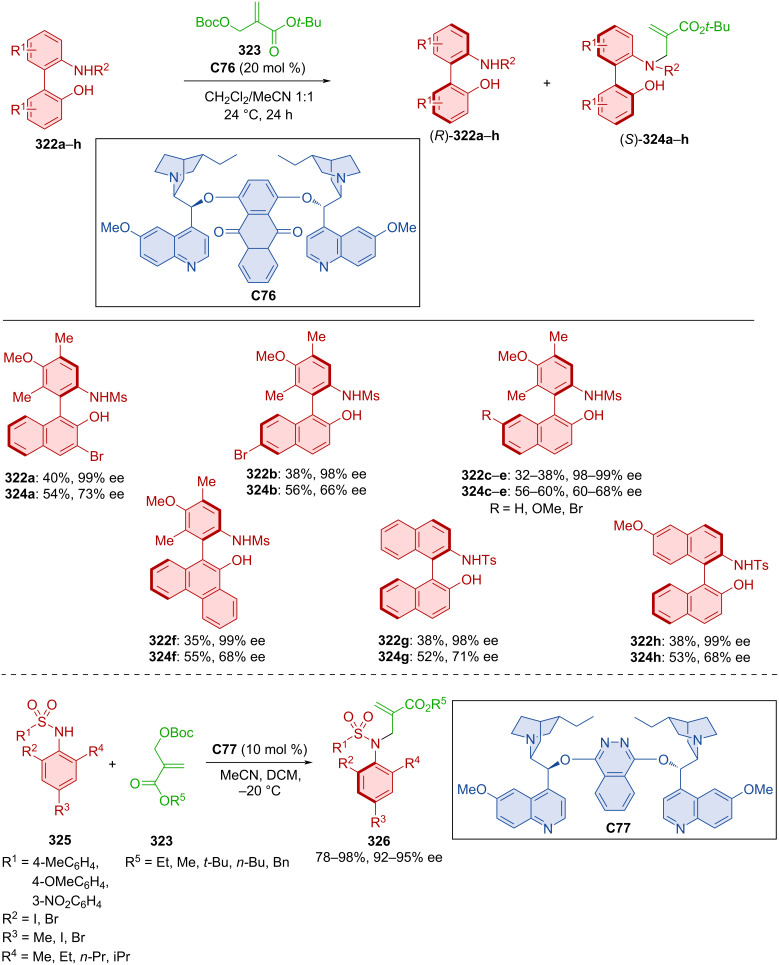
Atroposelective allylic substitution via kinetic resolution of biarylsulfonamides.

Lu et al. developed kinetic resolution by *N*-alkylation reaction of racemic biaryls **322a**–**h** with carbonate **323** catalyzed by dimeric hydroquinone organocatalyst **C76** (Scheme 98) [142]. *N*-Alkylated axially chiral products (*S*)-**324a**–**h** were prepared with moderate to good enantiomeric purities, and enantioenriched products (*R*)-**322a**–**h** were prepared in almost enantiopure form. The organocatalyst **C76** was successfully regenerated after the reaction and could be readily used again with comparable efficiency. During the study of the reaction conditions, in some cases, the authors observed the formation of tertiary sulfonamides as two different isomers, with their ratios changing over time. These isomers were defined as atropoisomers of alkylated arylsulfonamides. Expanding on this methodology, axially chiral products **326** were prepared starting from arylsulfonamides **325** and carbonates **323** mediated by dimeric hydrochinone **C77**. Di-*ortho*-substituted substrates were utilized, providing a significantly higher rotational barrier than mono-*ortho*-substituted ones. The optimized reaction conditions led to the formation of a considerable number of *N*-alkylarylated sulfonamides **326** with excellent yields and enantiomeric purities.

**Scheme 99:**
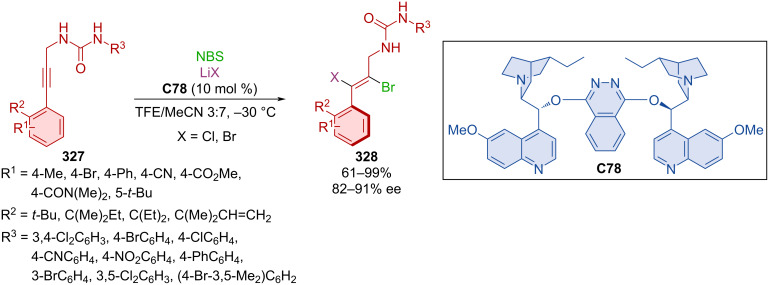
Atroposelective bromo-functionalization of alkynylarenes.

Wu et al. primarily focused on diastereoselective halogenations of double bonds [143]. In the process of substrate scoping they also developed a protocol, where halogenation happens on the triple bond of a suitable substrate **327** with the help of hydroquinine-based organocatalyst **C78** leading to axially chiral products **328** (Scheme 99). By this approach decent to near perfect yields and very good enantioselectivities were achieved.

**Scheme 100:**
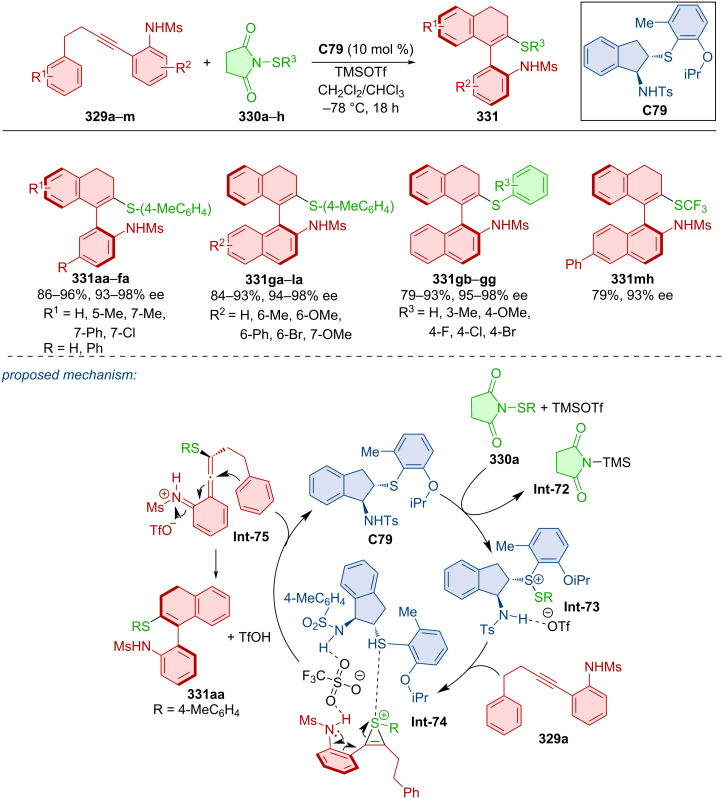
Sulfenylation-induced atroposelective cyclization.

Liang et al. constructed axially chiral amino sulfide vinyl arenes **331** by cyclization of alkynylanilines **329a**–**m** and electrophilic sulfur reagents **330a**–**h** in the presence of TMSOTf and organocatalyst **C79** (Scheme 100) [144]. This approach led to a wide range of axially chiral products in high yields and high degrees of enantiomeric purity. The thio and amino groups were subject to further transformations. For example, sulfur oxidation to sulfonyl, followed by substitution and reduction or deprotection of the amine, and diazotization can be done with subsequent bromination. The plausible reaction mechanism starts by the activation of the electrophilic sulfur reagent **330a** by organocatalyst **C83** and the Lewis acid. Thiirenium ion intermediate **Int-74** is formed with **329a** and by further conversion through aza-vinylidene-quinone-methide (aza-VQM) intermediate **Int-75**. Cyclization and rearomatization then afford the axially chiral product **331aa**.

An interesting report of catalysis by ketoreductase (KRED) for the preparation of atropoisomers was published in 2023 [145]. DKR of *N*-arylindolecarbaldehydes provided axially chiral products with consistently good yields and high levels of enantiomeric purities. The model product was successfully incorporated into a bifunctional thiourea organocatalyst.

**Scheme 101:**
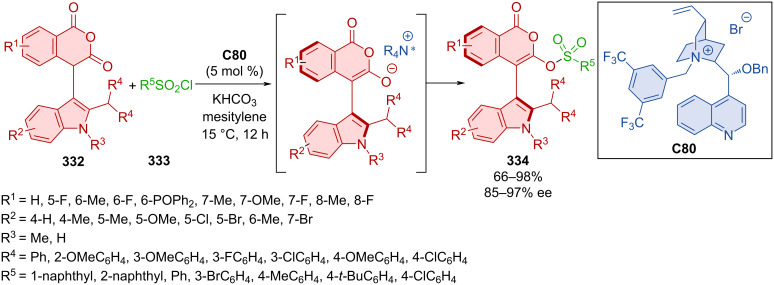
Atroposelective *O*-sulfonylation of isochromenone-indoles.

The cinchona alkaloid-derived phase-transfer chiral organocatalyst **C80** was utilized in dynamic kinetic resolution by sulfonylation of isochromenone-indoles **332** with sulfonyl chlorides **333** (Scheme 101) [146]. Axially chiral isochromenone-indoles **334** were prepared in moderate to excellent yields with a high degree of enantiomeric purity. Reaction mechanism studies suggest that the key reaction step responsible for the stereocontrol happens when the axially chiral enolate ion forms an ionic pair with the quaternary ammonium salt. This activated complex then forms the corresponding enantiomeric product faster, than the opposite one. Configurational stability was determined after stirring one of the products **334** in isopropanol at 110 °C for 9 h with no erosion of the enantioselectivity. The rotational barrier was theoretically calculated to be 39.7 kcal/mol, which defines it as class-3 atropoisomer.

**Scheme 102:**
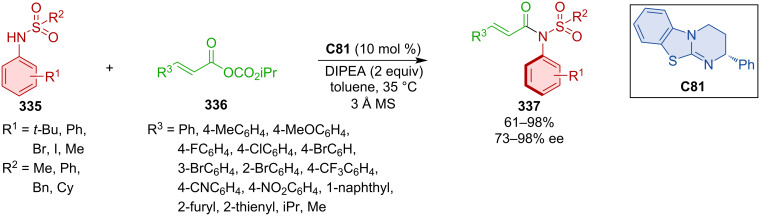
NHC-catalyzed atroposelective *N*-acylation of anilines.

Compounds with a C–N stereogenic axis are an interesting type of axially chiral derivatives. Dong and co-workers developed an atroposelective *N*-acylation of aniline-derived sulfonamides **335** (Scheme 102) [147]. The reaction afforded a range of sulfonyl-substituted anilide products **337** in good yields and high enantiomeric purities.

**Scheme 103:**
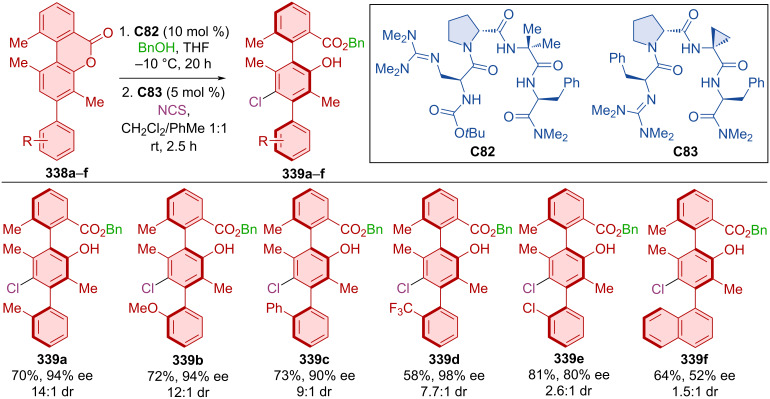
Peptide-catalyzed atroposelective ring-opening of lactones.

Beleh et al. presented a tandem dynamic kinetic resolution catalyzed by two different peptide organocatalysts **C82** and **C83** in 2020 (Scheme 103) [148]. Products of these subsequent transformations were axially chiral structures **339a**–**f** bearing two axially chiral axes. The first of the two reactions was the atroposelective lactone-ring opening of racemic polycyclic substrate **338a**–**f**, after which atroposelective chlorination of the central benzene ring took place. Despite the stereochemical complexity of the reaction, excellent enantioselectivities and moderately good yields were reported. A number of different approaches were tested, such as utilizing **C82** as a catalyst in both reactions and adding the chlorination reagent after the first twenty hours of reaction time. Decreased enantio- and diastereoselectivities were achieved by this method as opposed to standardized methodology (76% ee, 3.5:1 dr). Combining both organocatalysts and chlorination reagents from the get-go did not provide any product after 20 h. Lastly, by adding both organocatalysts initially and the chlorination reagent after 24 h, the product was formed with decent enantiomeric purity (84% ee, 5.5:1 dr), proving the superior efficiency of the tandem approach.

**Scheme 104:**
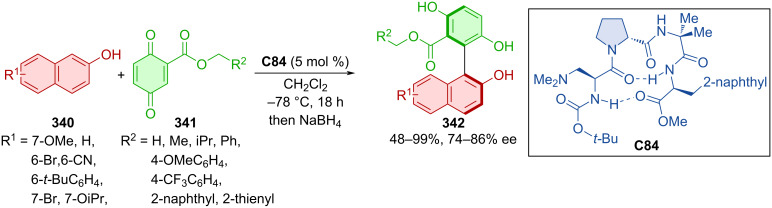
Peptide-catalyzed coupling of 2-naphthols with quinones.

Peptide **C84** was successfully utilized in the coupling reaction of 2-naphthols **340** with quinones **341** giving rise to the axially chiral naphthylhydroquinones **342** in moderate to excellent yields and good enantioselectivities (Scheme 104) [149]. A substrate containing a nitrile group in position 6 of the 2-naphthol deviates from otherwise decent yields, providing the product in only 48% yield. In a preparative scale reaction, the authors were able to achieve nearly enantiopure products after recrystallization, where significant product enantioenrichment happened after just one recrystallization cycle.

Similar peptide-catalyzed asymmetric cyclizations led to the formation of pharmaceutically relevant atropoisomeric *N*-arylquinazolinediones in mostly great yields, but with limited enantiomeric purity (70–97%, 32–84% ee) [150].

**Scheme 105:**
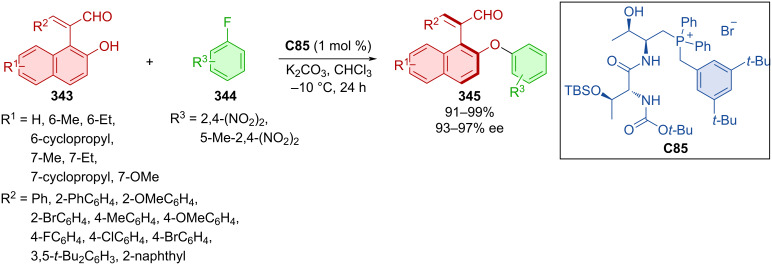
Atroposelective nucleophilic aromatic substitution of fluoroarenes.

In 2023, a unique chiral phosphonium salt **C85**-catalyzed reaction of alkenyl-2-naphthols **343** with fluorobenzenes **344** forming axially chiral products **345** was carried out (Scheme 105) [151]. With the optimized reaction conditions near perfect yields and enantioselectivities were reported.

## Conclusion

Axially chiral compounds rose from stereochemical peculiarities to diverse groups of compounds with many interesting properties and applications that range from catalysis, through materials to medicine. Atroposelective organocatalysis became one of the main methods for efficient synthesis of compounds chirality axes. Analysis of recent progress in this area has shown that all major organocatalytic activation modes are employed for realizing atroposelective transformations. From among covalent activation, classical enamine/iminium activation as well as NHC-catalyzed reactions were utilized to engage predominantly carbonyl-based transformations. The majority of recent organocatalytic reactions were catalyzed by non-covalent catalysts. Here the greatest share and the broadest diversity can be found in the chiral Brønsted acid catalysts. Interesting to note is also the fact that the most efficient structural motif for catalyst construction is actually an axially chiral binaphthylphosphoric acid and its congeners. From among other non-covalent organocatalysts, hydrogen-bond-donating thioureas and squaramides as well as other types of donors such alcohols or peptides were successfully employed in the stereoselective formation of axially chiral compounds. Several examples of phase-transfer-catalyzed reactions conclude the discussion. This review documents continuing development of this burgeoning area of research and documents tremendous creativity of our research community in assembling complex compounds with axial chirality. One can also note that some synthetic potential of all organocatalytic approaches was not yet utilized. For instance, methodologies utilizing peptide or carbonyl catalysis were not yet developed. Similarly, other non-covalent strategies such as those utilizing ion-paring catalysis, halogen bonds will hopefully also find use in the atroposelective organocatalysis. With this overview of recent progress of atroposelective organocatalytic reactions we hope to provide readers in various areas with an update of how this field develops and were new directions may lay.

## Data Availability

Data sharing is not applicable as no new data was generated or analyzed in this study.
